# Immunophenotype of Gastric Tumors Unveils a Pleiotropic Role of Regulatory T Cells in Tumor Development

**DOI:** 10.3390/cancers13030421

**Published:** 2021-01-23

**Authors:** Sara Rocha, Afonso P Basto, Marieke E Ijsselsteijn, Sara P Teles, Maria M Azevedo, Gilza Gonçalves, Irene Gullo, Gabriela M Almeida, Joaquín J Maqueda, Marta I Oliveira, Fátima Carneiro, João T Barata, Luís Graça, Noel F C C de Miranda, Joana Carvalho, Carla Oliveira

**Affiliations:** 1 i3S—Instituto de Investigação e Inovação em Saúde, Universidade do Porto, 4200-135 Porto, Portugal; srocha@ipatimup.pt (S.R.); sara.teles@cruk.cam.ac.uk (S.P.T.); maria.azevedo@ibmc.up.pt (M.M.A.); igullo@ipatimup.pt (I.G.); galmeida@ipatimup.pt (G.M.A.); jmaqueda@ipatimup.pt (J.J.M.); fcarneiro@ipatimup.pt (F.C.); jcarvalho@ipatimup.pt (J.C.); 2 Ipatimup—Institute of Molecular Pathology and Immunology of University of Porto, 4200-135 Porto, Portugal; 3 Doctoral Program on Cellular and Molecular Biotechnology Applied to Health Sciences, ICBAS—Instituto de Ciências Biomédicas Abel Salazar, Universidade do Porto, 4050-313 Porto, Portugal; 4 iMM—Instituto de Medicina Molecular João Lobo Antunes, Faculdade de Medicina da Universidade de Lisboa, 1649-028 Lisbon, Portugal; abasto@fmv.ulisboa.pt (A.P.B.); joao_barata@medicina.ulisboa.pt (J.T.B.); lgraca@medicina.ulisboa.pt (L.G.); 5 Instituto Gulbenkian de Ciência, 2780-156 Oeiras, Portugal; 6 Department of Pathology, Leiden University Medical Center, 2333 ZA Leiden, The Netherlands; M.E.IJsselsteijn@lumc.nl (M.E.I.); n.f.de_miranda@lumc.nl (N.F.C.C.d.M.); 7 Department of Pathology, Faculty of Medicine of the University of Porto (FMUP), 4200-319 Porto, Portugal; gilza.goncalves@lns.etat.lu; 8 Department of Pathology, Centro Hospitalar Universitário de São João, 4200-319 Porto, Portugal; 9 International Iberian Nanotechnology Laboratory, 4715-330 Braga, Portugal; marta.oliveira@inl.int

**Keywords:** tumor-infiltrating CD4 T cells, regulatory T cells, molecular regulation, gastric cancer

## Abstract

**Simple Summary:**

The role of regulatory T cells (Tregs) in gastric cancer (GC) is still controversial and poorly understood. GC patients have increased numbers of Tregs in peripheral blood and among tumor infiltrating lymphocytes; however, their prognostic value depends on specific tumor features (e.g., tumor location and/or microsatellite instability status). We found that Tregs might induce membrane expression of IL2Rα in intestinal-type GC cells, which associates with MAPK signaling pathway activation and spheroid growth. Moreover, Tregs accumulate at early steps of intestinal-type GCs progression, when tumors are starting to grow through the stomach wall, and do not present vascular and perineural invasion. Our findings suggest a novel non-immunosuppressive role of Treg cells in intestinal-type GC, which may unlock novel therapeutic immuno-oncology strategies for intestinal-type GC or other tumors with similar immune context.

**Abstract:**

Gastric cancer (GC) patients display increased regulatory T cell (Tregs) numbers in peripheral blood and among tumor-infiltrating lymphocytes. Nevertheless, the role of Tregs in GC progression remains controversial. Here, we sought to explore the impact of Tregs in GCs with distinct histology, and whether Tregs can directly influence tumor cell behavior and GC development. We performed a comprehensive immunophenotyping of 82 human GC cases, through an integrated analysis of multispectral immunofluorescence detection of T cells markers and patient clinicopathological data. Moreover, we developed 3D in vitro co-cultures with Tregs and tumor cells that were followed by high-throughput and light-sheet imaging, and their biological features studied with conventional/imaging flow cytometry and Western blotting. We showed that Tregs located at the tumor nest were frequent in intestinal-type GCs but did not associate with increased levels of effector T cells. Our in vitro results suggested that Tregs preferentially infiltrated intestinal-type GC spheroids, induced the expression of IL2Rα and activation of MAPK signaling pathway in tumor cells, and promoted spheroid growth. Accumulation of Tregs in intestinal-type GCs was increased at early stages of the stomach wall invasion and in the absence of vascular and perineural invasion. In this study, we proposed a non-immunosuppressive mechanism through which Tregs might directly modulate GC cells and thereby promote tumor growth. Our findings hold insightful implications for therapeutic strategies targeting intestinal-type GCs and other tumors with similar immune context.

## 1. Introduction

Recent advances on the molecular aspects of gastric cancer (GC) have provided invaluable knowledge that led to the identification of new actionable targets and therapies [1]. Targeted treatments and immunotherapies are mainly offered to advanced cancer patients and have, so far, minimally improved GC prognosis, justifying why GC ranks as the third leading cause of cancer-related deaths worldwide [2]. Gastric tumors with high mutation load, microsatellite instability (MSI) or positive for Epstein–Barr viral infection are potentially immunogenic, and thus amenable for immunotherapy based on checkpoint inhibitors (e.g., anti- PD-1/PD-L1) [3,4]. Finding additional contexts for immuno-related therapies is an opportunity that is worth investigating further.

Regulatory T cells (Tregs) are CD4^+^ T cells characterized by the surface expression of IL2Rα, and nuclear expression of the transcription factor FoxP3. Tregs are endowed with immunosuppressive activity that enforces peripheral tolerance and maintains immunological homeostasis [5]. However, in cancer, the immunosuppressive environment promoted by Tregs withholds the antitumor immune response, hence promoting tumor progression and dissemination [6,7]. In GC patients, peripheral blood and tumor-infiltrating lymphocytes are enriched in Tregs [8,9,10,11,12], which associates with increased tumor stage, poor prognosis and reduced patient survival [13,14,15,16,17]. Nevertheless, other studies have shown that tumor infiltrating Tregs may also be associated with favorable prognosis, specifically in patients carrying tumors from the cardia or with MSI [18,19,20]. Hence, the role of Tregs in GC progression remains poorly understood and highly controversial. Further, it is also unexplored in GC whether Tregs may promote tumor progression via non-immunological mechanisms, as described in other cancer models [21,22]. Thus, we sought to explore the contribution of Tregs in GCs with distinct histology, and whether Tregs can directly impact tumor cells to promote GC progression.

To address this, we integrated the results of T cell immunophenotyping analysis with clinicopathological features of 82 GC patients. This analysis unveiled an enrichment of Tregs specifically in intestinal- and indeterminate-type GC, as compared to diffuse-type GC. Furthermore, our data suggests that a population of Tregs is present at the tumor nest of intestinal-type GC independently of the prevalence of effector T cells. Given these results, and to understand whether Tregs may actively modulate the phenotype of tumor cells, we established and explored 3D co-cultures of Tregs with intestinal- or diffuse-type GC cell lines. We found that Tregs actively infiltrate intestinal-type GC spheroids. Upon co-culture with Tregs, intestinal-type GC cells acquire expression of IL2Rα at the cell membrane, have increased activation of MAPK signaling pathway and spheroid growth. Furthermore, we found an enrichment of Tregs in early-stage intestinal-type GC, and in the absence of vascular and perineural invasion.

Altogether, our data suggests a direct effect of Tregs on tumor cells that may be particularly important in early stages of intestinal-type GC progression.

## 2. Results

This section may be divided by subheadings. It should provide a concise and precise description of the experimental results, their interpretation as well as the experimental conclusions that can be drawn.

### 2.1. Intestinal-Type GC Maintain a Population of Tregs at the Tumor Nest Independently on the Prevalence of Effector T Cells

**Figure 1:**
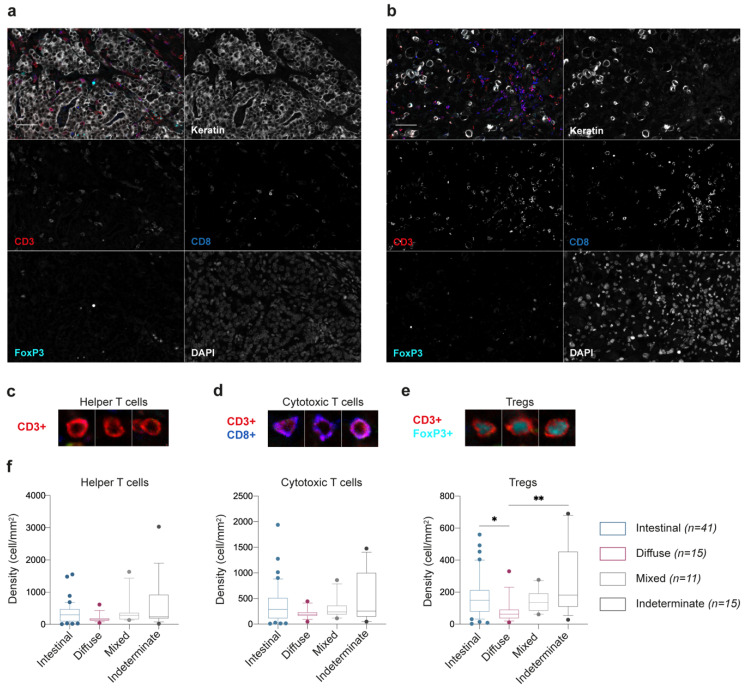
Characterization of the immune T cell landscape in gastric cancer (GC) tissue sections. (**a**,**b**) Representative composite and single-staining immunofluorescence images of the two main histological GC types, intestinal-type (**a**) and diffuse-type (**b**), obtained by multispectral imaging. Scale bar: 100 μm. (**c**–**e**) Main T cell populations identified in the GC microenvironment based on the positivity for CD3, CD8, and FoxP3 cell markers. (**f**) Relative numbers of helper, cytotoxic T cells and Tregs, according to the histologic properties of the tumor: intestinal-type, *n* = 41; diffuse-type, *n* = 15; mixed-type, *n* = 11; indeterminate-type, *n* = 15 patients. Box and whiskers represent median ± 10 to 90 percentile. * *p* < 0.05, ** *p* < 0.01; Kruskal–Wallis test with Dunn’s multiple comparison test.

**Figure 2:**
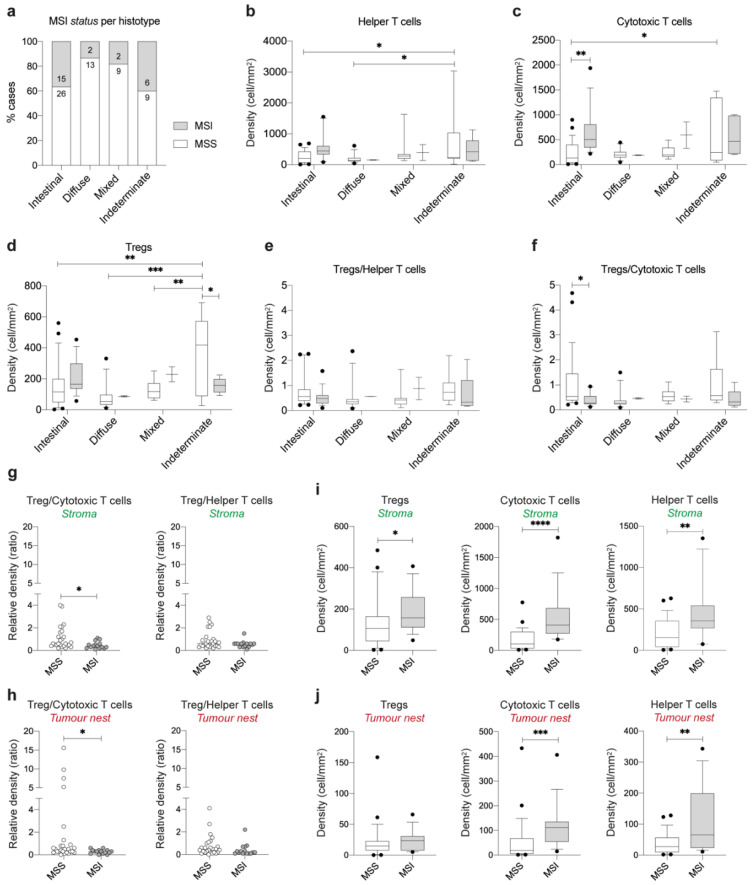
Distribution of T cell populations in MSS and microsatellite instability (MSI) GC tumors. (**a**) Relative and absolute frequency of MSS and MSI cases for each GC histological type. (**b**–**f**) Relative numbers of helper (**b**), cytotoxic T cells (**c**), Tregs (**d**), and Tregs normalized to helper T cells (**e**) and cytotoxic T cells (**f**), according to the MSI status and tumor histology. (**g**,**h**) Relative number of Tregs normalized to cytotoxic and helper T cells within the stroma (**g**) and tumor nest (**h**) areas of intestinal-type GC cases, according to MSI status. Relative number of Tregs, cytotoxic T cells, and helper T cells within the stroma (**i**) and tumor nest (**j**) areas of intestinal-type GC cases, according to MSI status. See Supplementary Figure S1 for a visual description of stroma and tumor nest areas. Box and whiskers represent median ± 10 to 90 percentile. * *p* < 0.05, ** *p* < 0.01, *** *p* < 0.001, **** *p* < 0.0001. Two-way ANOVA with Tukey’s/Sidak’s multiple comparisons test (**b**–**f**) and Mann–Whitney *U* test (**g**–**j**).

To investigate the immune T cell landscape within GC, we performed immunophenotyping of 82 patient samples. Each tumor section was stained simultaneously for a panel of seven markers, including CD3, CD8, and FoxP3 T cell-associated markers, cytokeratin to label epithelial tumor cells and DAPI for nuclei detection (Figure 1a,b). We focused the analysis on three major phenotypes, defined as follows: (1) Helper T cells, CD3^+^CD8^-^FoxP3^-^ cells (Figure 1c); (2) Cytotoxic T cells, CD3^+^CD8^+^ cells (Figure 1d); and (3) Tregs, CD3^+^CD8^-^FoxP3^+^ cells (Figure 1e). Cells expressing cytokeratin were excluded when building the T cell profiles. Poor performance of anti-IL2Rα antibodies in the immunofluorescence staining hampered the analysis of IL2Rα expression in tissue samples.

To disclose a potential association between T cell phenotypes and GC histology, we evaluated the density of each cell population according to GC histological types (Figure 1f; Supplementary File S1). We observed that whereas the presence of helper and cytotoxic T cells only slightly changed across GC histological types, Tregs were significantly enriched in intestinal- and indeterminate-type GC, as compared to diffuse-type GC (*p* = 0.0175 and *p* = 0.0028, respectively; Figure 1g).

We further assessed whether these differences could be explained through a differential prevalence of MSI cases within each histotype. Whilst 37% and 40% of intestinal- and indeterminate-type GC cases were MSI, only 13% and 18% of diffuse- and mixed-type GC had this phenotype (Figure 2a). Given these differences, we re-assessed the distribution of T cell populations considering both GC histotype and MSI status (Figure 2b–f). We found that indeterminate-type MSS cases have more helper, cytotoxic cells and Tregs as compared to intestinal-type MSS cases (*p* = 0.0261, *p* = 0.0349 and *p* = 0.0026, respectively); display increased numbers of helper T cells and Tregs in comparison to diffuse-type MSS (*p* = 0.0297 and *p* = 0.0002, respectively); and increased number of Tregs in comparison to mixed-type MSS (*p* = 0.0065; Figure 2b–d). Although, indeterminate-type MSS cases had increased number of Tregs as compared to MSI cases (*p* = 0.0444; Figure 2d), the Treg/cytotoxic T cell and Treg/helper T cell ratios were not significantly changed (Figure 2e,f). Overall, these results suggested that indeterminate-type MSS cases elicit stronger immune responses, as compared to the other histotypes, and that the increased accumulation of Tregs was likely the result of an accumulation of effector T cell populations. As for intestinal-type GC, MSS cases displayed significantly lower numbers of cytotoxic T cells (*p* = 0.0014), but not of Tregs, as compared to MSI cases (Figure 2c,d). This led to a higher Treg/cytotoxic T cell ratio in intestinal-type MSS cases comparing with MSI cases (*p* = 0.0414; Figure 2f).

To further dissect this unbalanced ratio in intestinal-type GC, we characterized the distribution of T cell populations regarding their location at the stroma or tumor nest areas (Figure 2g–j; Supplementary Figure S1). We observed that the increased Treg/cytotoxic T cell ratio in MSS tumors was maintained both at the stroma and tumor nest regions (*p* = 0.0274 and *p* = 0.0109, respectively; Figure 2g,h). Of notice, several MSS intestinal-type tumors showed particularly high numbers of Tregs comparing to the numbers of cytotoxic T cells, at the tumor nest (Figure 2h). Moreover, we observed that the density of Tregs was comparable at the tumor nest of MSS and MSI intestinal-type GCs, while the density of cytotoxic T cells was only increased at the tumor nest of MSI tumors (Figure 2i,j). These observations may indicate that the cytotoxic T cell density is not being accompanied by an increased Treg density at the tumor nest, as observed for the stroma region. This data further suggests that both MSS and MSI cases have a population of Tregs, in the close vicinity of tumor cells, that does not seem to have a purely immunosuppression role, as their density does not accompany the increase in cytotoxic T cells.

Altogether, these observations support the hypothesis that Tregs may have a non-immunosuppressive activity by directly affecting GC tumor cells with intestinal histology.

### 2.2. Tregs Actively Infiltrate Intestinal-Type GC Spheroids

To scrutinize the potential interactions between Tregs and tumor cells, we established direct 3D in vitro co-cultures of T cells, isolated from the peripheral blood of healthy donors, with GC cell lines (intestinal-type MKN74, and diffuse-type MKN45). Given the impossibility of using intracellular FoxP3 for cell sorting, we isolated CD3^+^CD4^+^CD127^−^IL2Rα^+^ T cells, which are highly enriched in Tregs, from the peripheral blood of healthy donors (Supplementary Figure S2). As a control, CD3^+^CD4^+^CD127^+^IL2Rα^−^ cells (conventional CD4 T cells) were also collected from the same donors.

**Figure 3:**
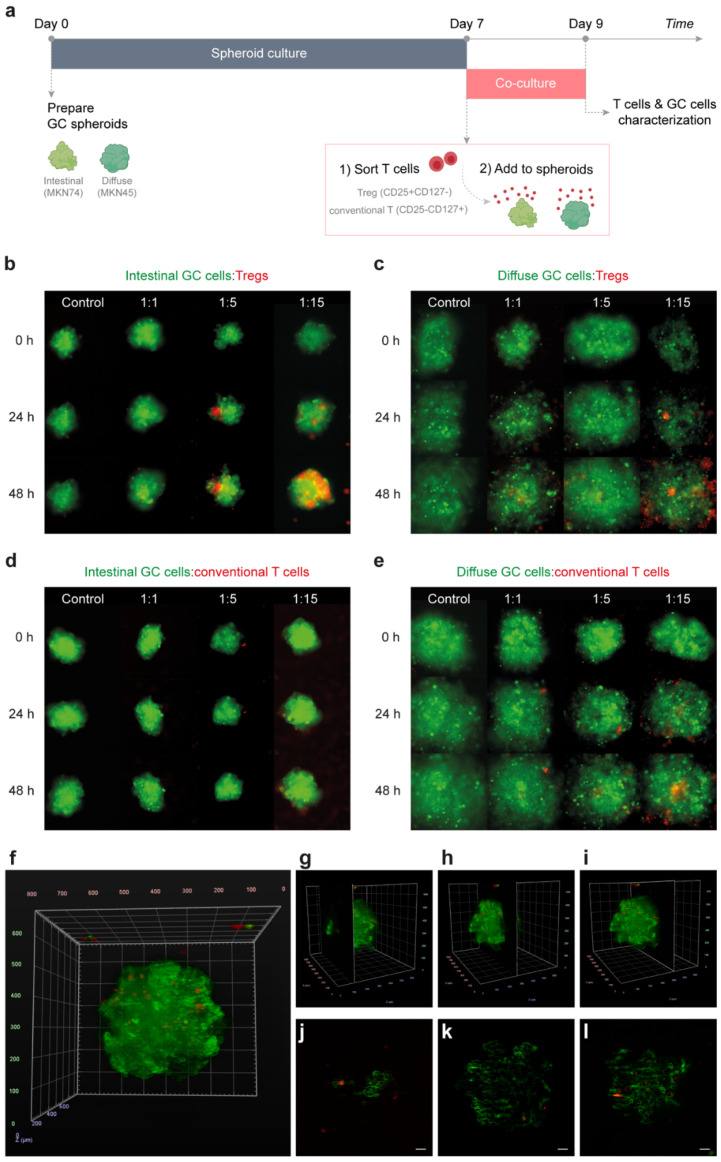
T cell infiltration of GC spheroids. (**a**) Schematic representation of the co-culture timeline. Spheroids (green) resembling intestinal- and diffuse-type GC were independently cultured for 7 days. Thereafter, Tregs and conventional T cells (red) were sorted from peripheral blood of healthy donors, based on the expression of CD3, CD4, IL2Rα, and CD127 T cell markers, and added to GC spheroids. After 48 h of co-culture, spheroids were dissociated for further characterization of both T cells and GC cells. (**b**–**e**) Monitorization of GC spheroids-T cell interactions during the 48 h of co-culture by time-lapse microscopy. Control represents GC spheroids (green) that were not cultured with T cells (red). 1:1, 1:5 and 1:15 (GC cell:T cell) represent the increasing proportions of T cells to GC cells tested for (**b**) intestinal-type spheroids co-cultured with Tregs, (**c**) diffuse-type spheroids co-cultured with Tregs, (**d**) intestinal-type spheroids co-cultured with conventional T cells, (**e**) diffuse-type spheroids co-cultured with conventional T cells. (**f**,**g**) Light-sheet microscopy of 24 h co-cultures of intestinal-type GC spheroids (green) and Tregs (red). (**f**) Co-culture 3D visualization after four-angle fusion of light-sheet microscopy images. (**j**–**l**) Lateral views of the 3D representation obtained from the light-sheet imaging data corresponding to the regions indicated in (**g**) ~124 nm, (**h**) ~290 nm, and (**i**) ~434 nm, to the entire co-culture. Scale bar: 50 μm.

T cells (either Tregs or conventional T cells) were added to GC spheroids recapitulating intestinal- or diffuse-type GC (Figure 3a). Co-cultures, imaged with time-lapse for 48 h, evidenced distinct Treg infiltration capacities depending on GC histology (Figure 3b,c). Whilst Tregs accumulated inside the intestinal-type GC spheroids (Figure 3b), they remained preferentially at the periphery of diffuse-type spheroids (Figure 3c). In both situations, the accumulation of Tregs was proportional to the number of Tregs in the co-culture (Figure 3b,c). As far as conventional T cells are concerned, accumulation either inside or at the periphery of spheroids was very low as compared to that of Tregs (Figure 3d,e). These observations support the findings from patients’ tumors and reinforce specific crosstalk between Tregs and intestinal-type GC cells. Active infiltration of intestinal-type GC spheroids by Tregs was further validated through a four-angle analysis of the spheroids at distinct time-points (Figure 3f–l; Supplementary Figure S3; Supplementary Videos S1–S3). This analysis revealed that only a small fraction of Tregs was actively infiltrating the spheroids (Figure 3f–l), recapitulating the proportion of Treg infiltration observed in patients’ intestinal tumors (Figure 1a).

### 2.3. Tregs Induce IL2Rα Expression at the Membrane of Intestinal-Type GC Cells

**Figure 4:**
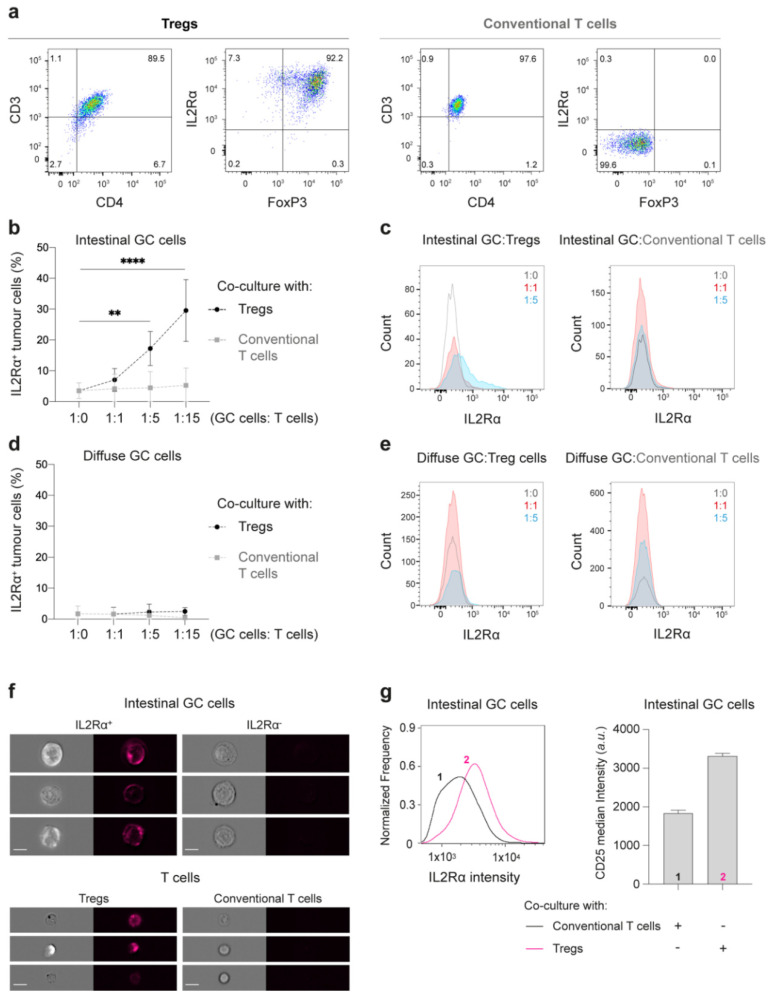
Characterization of GC and T cells after 48 h of co-culture. (**a**) Representative pseudocolor plots with the expression of CD3, CD4, IL2Rα, and FoxP3 T cells markers by flow cytometry show that Tregs and conventional T cells maintain their characteristic phenotype after 48 h of co-culture. (**b**–**e**) Quantitative expression of IL2Rα in GC cells. (**b**,**d**) Plots showing percentage of IL2Rα positive intestinal- (**b**) or diffuse-type GC (**d**) cells after 48 h co-culture with Tregs (black) or conventional T cells (grey). Data are shown as mean ± SD for co-cultures treated with T cells isolated from at least six healthy donors. ** *p* < 0.01, **** *p* < 0.0001. Two-way ANOVA with Dunnett’s multiple comparisons test. (**c**,**e**) Representative histograms of IL2Rα expression in intestinal- (**c**) or diffuse-type GC (**e**) cells after co-cultured at 1:1 (red) or 1:5 (blue) proportions with T cells. (**f**,**g**) Detection of membranous IL2Rα expression by imaging flow cytometry. (**f**) Representative images of IL2Rα expression in IL2Rα positive (top left) and negative (top right) intestinal GC cells, as well as in Tregs (bottom left) and conventional T cells (bottom right). Scale bar: 10 μm. (**g**) Representative histogram of IL2Rα expression at the cell membrane (left graph) of intestinal-type GC cells co-cultured with conventional T cells (1-dark line) or Tregs (2-pink line) at 1:5 proportion and after 48 h of co-culture. Quantification of the median IL2Rα intensity at the cell membrane (right graph). Graphs represent data from at least three independent experiments.

Next, we investigated whether GC cells could affect the phenotype of T cells and vice-versa. We started by analyzing the expression of CD3, CD4, IL2Rα, and FoxP3 T cell markers in cells collected from the co-culture conditioned media, and after dissociation of GC spheroids into single-cell suspensions. To assess the expression of these markers both in T cells (Tregs and conventional, stained with CTV) and in GC cells, we followed the gating strategy showed on Supplementary Figure S4. Both Tregs and conventional T cells maintained their original phenotype after 48 h of co-culture (Figure 4a), indicating that GC cells unlikely impact the expression of markers of Treg activation and immunosuppression, such as IL2Rα and FoxP3. Surprisingly, when co-culturing intestinal-type GC cells with Tregs, but not with conventional T cells, GC cells show de novo expression of membranous IL2Rα (Figure 4b,c), the α-chain of the IL2 receptor. This phenomenon was specific for intestinal-type GC cells, and the extent of expression was proportional to the number of Tregs in the co-culture (Figure 4b–e). When intestinal-type GC spheroids were cultured in media supplemented with IL2 and increasing concentrations of anti-CD3/anti-CD28 beads, no IL2Rα induction was observed (Supplementary Figure S5A). Moreover, conditioned media from co-cultures did not elicit IL2Rα expression in tumor cells from spheroids that have not been previously exposed to T cells (Supplementary Figure S5B,C).

We further confirmed by imaging flow cytometry, that after co-culture with Tregs, intestinal-type GC cells displayed a 1.8-fold increase of the median membranous IL2Rα fluorescence intensity (3312 ± 72 a.u.), in comparison to GC cells cultured with conventional T cells (1839 ± 71 a.u.; Figure 4f,g).

Altogether, these results suggest that while GC cells do not seem to affect the phenotype of Tregs, their direct interaction induces expression of IL2Rα at the membrane of intestinal-type GC cells.

### 2.4. IL2Rα Expression in Intestinal-Type GC Cells Associates with MAPK Signalling Pathway Activation and Spheroid Growth

**Figure 5:**
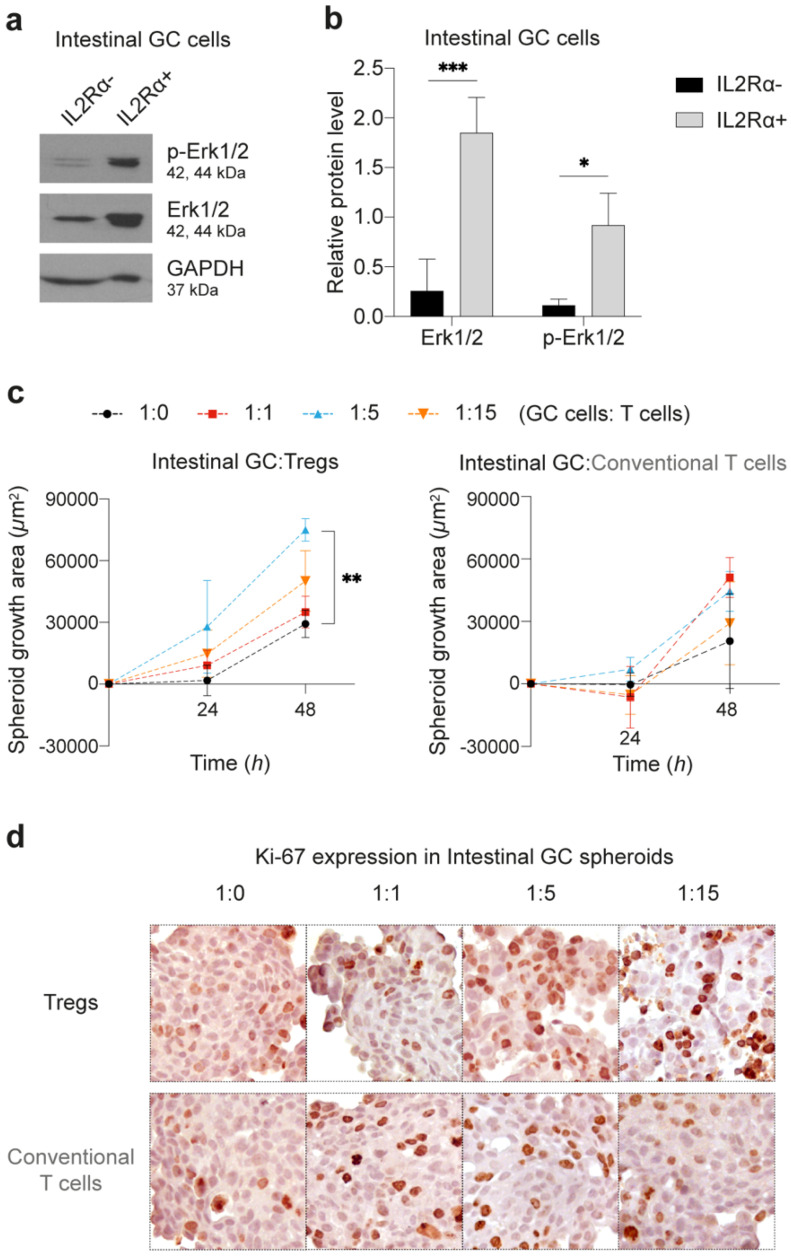
Spheroid growth after co-culture with T cells. (**a**,**b**) Western blot analysis for phosphorylated (p-ERK1/2) and total ERK1/2 protein levels in intestinal-type IL2Rα positive and negative cells after 48 h co-culture with Tregs (1:5 proportion). (**a**) WB scans and (**b**) normalized expression represent three independent experiments. (**c**) Plots of growth area for intestinal GC spheroids co-cultured with Tregs (left graph) or conventional T cells (right graph) for 48 h. Control spheroids (dark) represent intestinal-type GC spheroids without T cells in co-culture. Co-cultures at 1:1, 1:5, and 1:15 proportions are represented in red, blue, and orange, respectively. Data shown mean ± SD of three independent co-cultures. * *p* < 0.05, ** *p* < 0.01, *** *p* < 0.001. Two-way ANOVA with Dunnett’s multiple comparisons test. (**d**) Representative images of Ki-67 nuclear expression (dark brown nuclei) in intestinal-type GC spheroids after 48 h of co-culture with Tregs or conventional T cells, at different 1:0 (control), 1:1, 1:5, or 1:15 proportions.

IL2 is a key cytokine in the regulation of immune cell activation and proliferation, particularly in Tregs and effector T cells. In immune cells, the high-affinity IL2 receptor, comprising the α-chain (CD25), β-chain (CD122) and γc-chain (CD132), initiates signal transduction via JAK1/3, leading to the activation of MAPK, PI-3K and STAT signaling pathways [23,24]. We tested whether these pathways were activated in intestinal-type GC cells expressing IL2Rα, since, to the best of our knowledge IL2Rα expression has never been described in epithelial tumor cells. We observed that sorted IL2Rα^+^ intestinal-type GC cells overexpressed total and phospho-ERK1/2 (Figure 5a,b; Supplementary Figure S6A), but STAT3 and Akt expression/activation were not detected (Supplementary Figure S6B). Accordingly, we observed that intestinal-type GC spheroids co-cultured with Tregs had increased growth, as a consequence of higher proliferation (*p* = 0.0021; Figure 5c,d; Supplementary Figure S6C). Indeed, during the first 24 h of co-culture, GC:Treg spheroid growth rate particularly increased in the 1:5 condition in comparison to control spheroids, and to GC:conventional T cell co-cultures (Figure 5c). This observation indicates an early proliferative effect of Tregs over intestinal-type GC cells. In contrast, Tregs, but not conventional T cells, induced a reduction of diffuse-type GC spheroid growth after 24 h of co-culture as compared to control spheroids (*p* < 0.05; Supplementary Figure S6D). The latter observations suggest that Tregs may have the opposite effect over diffuse-type GC spheroids, and likely through a distinct mechanism. Altogether, these results show that Tregs potentiate activation of the MAPK signaling pathway and intestinal-type GC spheroid growth.

### 2.5. Early-Stage Intestinal-Type GCs Are Enriched in Tregs

**Figure 6:**
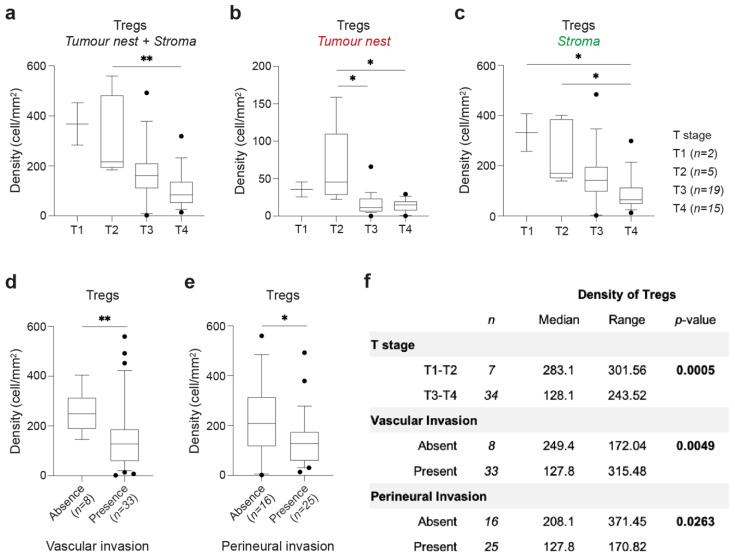
Distribution of Tregs in intestinal-type GCs and association with clinicopathological features. (**a**–**c**) Density of Tregs at tumor nest and stroma areas (**a**), tumor nest only (**b**), or stroma only (**c**), according to T stage: T1, *n* = 2; T2, *n* = 5; T3, *n* = 19; T4, *n* = 15. (**d**) Density of Tregs in intestinal-type GC with absence (*n* = 8) or presence (*n* = 33) of vascular invasion. (**e**) Density of Tregs in intestinal-type GC with absence (*n* = 16) or presence (*n* = 25) of perineural invasion. (**f**) Summary of the association of Treg density with clinicopathological features of intestinal-type GC. Box and whiskers represent median ± 10–90 percentile. * *p* < 0.05, ** *p* < 0.01; Kruskal–Wallis test with Dunn’s multiple comparison test (**a**–**c**) and Mann–Whitney U test (**d**–**f**).

Considering that Treg interaction with intestinal-type GC spheroids potentiates their growth, we hypothesized that the population of Tregs present at the tumor nest of intestinal-type GC could also trigger tumor growth. We addressed this hypothesis by analyzing whether the number of Tregs in GCs was associated with tumor growth into the stomach wall (T stage). We detected higher density of Tregs in earlier T stages of intestinal-type GC, namely comparing tumors limited to the submucosa and muscularis propria with those growing throughout the stomach wall and into nearby organs or structures (pT2 vs. pT4, *p* = 0.0092, Figure 6a; tumor nest: pT2 vs. pT3, *p* = 0.0263 and pT2 vs. pT4, *p* = 0.0119, Figure 6b; stroma: pT1 vs. pT4, *p* = 0.0493, and pT2 vs. pT4, *p* = 0.0305, Figure 6c). Additionally, a higher density of Tregs was also associated with the absence of vascular (*p* = 0.0049; Figure 6d) and perineural invasion (*p* = 0.0263, Figure 6e). Importantly, none of these associations were found when analyzing helper or cytotoxic T cells (Supplementary Figure S7A–C). Further, no association of T cell density was found with the presence/number of lymph nodes metastases (N), the presence of distant metastases (M), or with the TNM stage (Supplementary Figure S7D–F).

These data suggest that the crosstalk between Tregs and intestinal-type GC cells might be important at the early stages of tumor progression when the tumor is growing through the stomach wall into the muscularis propria, and when no vascular or perineural invasion is observed (Figure 6f).

## 3. Discussion

**Figure 7:**
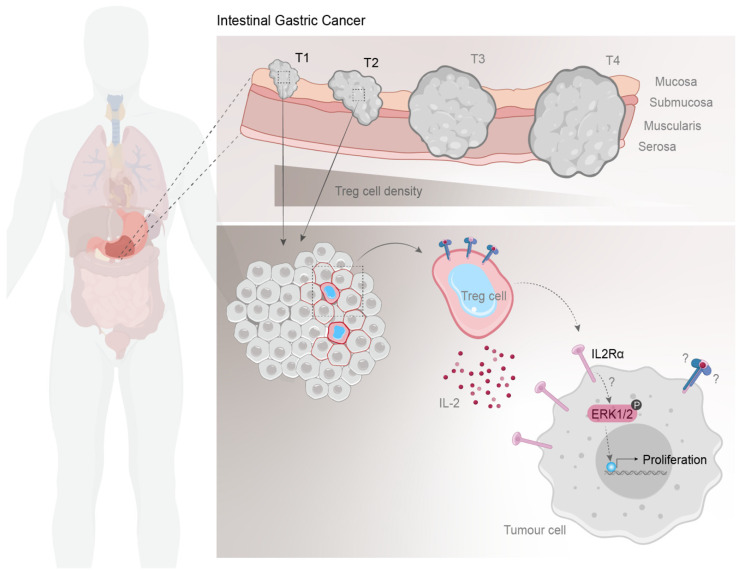
Schematic model of Treg prevalence in intestinal-type GC. Early-stage intestinal GC, that do not present vascular and perineural invasion, display higher density of Tregs. In vitro results indicate that Tregs might induce IL2Rα expression at the membrane of tumor cells, which might associate with activation of MAPK signaling pathway, increased proliferation, and spheroid growth. Further studies are required to understand the mechanism behind IL2Rα expression in tumor cells and its implications in future IO therapies.

In this study, we combined single-cell immunophenotyping of GC samples, clinicopathological data, and 3D in vitro co-culture cell models to propose a tumor-promoting, non-immunosuppressive role of Tregs in GC progression (Figure 7). According to our findings, intestinal-type GCs are enriched in Tregs that preferentially accumulate during the initial steps of tumor progression. Additionally, our in vitro results suggest that Tregs may promote proliferation of intestinal-type GC cells by inducing membranous expression of IL2Rα in tumor cells, which associated with MAPK pathway activation and cell proliferation. Together, these results led us to hypothesize that Tregs that successfully infiltrate the tumor nest of intestinal-type GCs may directly contribute to their well-known proliferative behavior.

Despite growing evidence of Treg enrichment in GC microenvironment, the role of Tregs in GC remains poorly understood. Studies showing that Tregs are already increased in non-neoplastic lesions, such as gastritis and peptic ulcers [25,26], suggest that Treg accumulation may be a consequence of inflammation and occurs before GC initiation. These findings have initially fostered our hypothesis that Tregs might differently contribute to the neoplastic development of tumors arising in distinct inflammatory contexts. To investigate this and considering recent data on the importance of studying the immunophenotype of GC [27,28], we performed a single-cell spatial analysis of the immune landscape of gastric tumors with distinct histology. Our results showed that among the T cell populations analyzed, Tregs were enriched in intestinal-type and indeterminate-type GC, which are often associated to chronic inflammation, in comparison to diffuse-type GC. This association of Tregs with inflammatory environments supports the proposed mechanisms of Treg accumulation in GC tumors based on CCL17/CCL22-mediated recruitment of Tregs or TGF-β-mediated conversion of conventional T cells into Tregs [29,30,31]. Another similarity between intestinal-type and indeterminate-type GCs was the increased proportion of MSI cases, which could elicit stronger anti-tumor immune responses [32]. Indeterminate-type MSS GCs showed elevated, though highly variable, T cell numbers that likely recapitulate the heterogenous nature of these tumors. Interestingly, in intestinal-type GCs, we observed an unbalanced ratio of Tregs and cytotoxic T cells, comparing MSS and MSI tumors. Such findings were more evident at the tumor nest of MSS tumors, where the number of Tregs was higher than the number of cytotoxic T cells. This excess of Tregs at the tumor nest fostered the hypothesis of a direct crosstalk between Tregs and intestinal-type tumor cells. Hence, we established direct co-cultures of T cells and 3D GC spheroids recapitulating the histological properties of intestinal- and diffuse-type GC [33]. This valuable tool allowed us to assess specific and active spheroid infiltration by T cells. As tumors can remodel their microenvironment, modulating Tregs’ phenotype or even converting conventional T cells into Tregs [7], we prepared co-cultures with T cells isolated from healthy donors, presumably without prior contact with tumor cells. We demonstrated that only a small fraction of Tregs infiltrates GC spheroids, even if high numbers of Tregs are added to the culture. Our 3D co-culture system not only closely mimics tumors from GC patients, but also their microenvironment context: (1) Treg density is higher outside than inside GC spheroids, which resembles the high T cell density at the GC stroma comparing to tumor nest; (2) Tregs, but not conventional T cells, infiltrate GC spheroids, recapitulating the increased Treg numbers observed in the tumor nest; (3) Tregs preferentially infiltrate intestinal-type GC spheroids, mimicking the histological differences observed between intestinal- and diffuse-type GC. Noteworthy is also the fact that intestinal-type GC spheroids display stronger homotypic cell-to-cell adhesion than diffuse spheroids [33], which would hamper Treg infiltration. Nevertheless, we also recognize the limitations to this approach. In this study, Tregs, but not conventional T cells, were stimulated with anti-CD3/anti-CD28 beads to maintain cell activation. A missing control of activated conventional CD4+ T cells is a significant limitation of this study as it would have allowed us to directly assess whether the observed effect is Treg-specific or driven by the stimulation of cells. Further studies should include additional and more relevant biological controls. Moreover, our co-culture system represents a simplistic representation of the in vivo scenario that should be further integrated with patient-derived xenografts or relevant in vivo models.

Our study suggests that the interaction between Tregs and tumor cells may have functional consequences, as intestinal-type cells acquire IL2Rα expression at the cell membrane, have stimulation of MAPK signaling pathway and increased spheroid growth. To our knowledge, this is the first study reporting IL2Rα de novo expression in epithelial tumor cells, which may hold important implications for the design of novel therapeutic strategies. Thus, it is critical to further understand the mechanisms that lead to IL2Rα expression in tumor cells and its molecular consequences. Our data suggests that IL2Rα is not passively transferred from Tregs to tumor cells, as tumor cells do not acquire other membrane Treg markers nor the cell tracer that was used to stain Tregs. Moreover, the conditioned medium of the Treg-intestinal tumor cell co-cultures was not sufficient to induce IL2Rα expression in other intestinal-type GC cells. Thus, it is unlikely that Treg-derived soluble factors or extracellular vesicles could actively foster or transfer IL2Rα to tumor cells. We expect that further studies focused on direct cell-cell interactions between Tregs and tumor cells, will enlighten the mechanisms underlying IL2Rα expression in tumor cells. Furthermore, it will be important to understand whether IL2Rα expression in tumor cells may also be induced by other activated T cell populations, as in this study conventional T cells were not activated.

In immune cells, IL2Rα is known to bind IL2 and form heterodimers with the β- (CD122) and γc- (CD132) chains of the IL2 receptor, to activate downstream signaling transduction [23,24]. Hence, it would be important to address whether IL2Rα-tumor cells additionally express CD122 and CD132, and are responsive to IL2 signals. These experiments are crucial to determine the molecular effects of Tregs in tumor cells that should be further validated in tissue samples. Although the results presented in this study suggest a novel and unexpected interaction between Tregs and tumor cells, the associated experimental limitations stress the need for further investigations to confirm the proposed hypothesis. Such experiments would require (1) the inactivation of IL2Rα and its downstream targets in tumor cells upon co-culture with Tregs; (2) a larger approach such as RNA sequencing or proteomics analysis on IL2Rα^+^ tumor cells to fully disclose the signaling pathways activated by IL2Rα expression; and (3) a quantitative demonstration that spheroid growth was due to enhanced tumor cell proliferation. This thorough analysis would provide key molecular targets that could be further validated in patient-derived samples.

A deeper understanding on why, and under which circumstances, Tregs may induce expression of IL2Rα in tumor cells, may greatly impact the therapeutic strategies for intestinal-type GC patients and others presenting tumors with similar behavior. This knowledge may also shed light on the contrasting effects of IL2-based tumor therapies [34]. Our results suggest that, under specific tumor contexts, therapies based on IL2 administration may favor tumor growth instead of just promoting immune T cell proliferation and survival. Additionally, tumor cells expressing IL2Rα at the membrane may compete with effector T cells for IL2, a tumor immune evasion strategy that, until now, was solely attributed to Tregs [7]. Finally, innovative tumor therapies may arise directly from knowing that the initial steps of tumor growth may be dependent/potentiated by IL2Rα-mediated signaling. Strategies targeting IL2Rα [35] can be specifically directed to epithelial cells, which may reduce the secondary effects from unspecific targeting of Tregs.

## 4. Materials and Methods

### 4.1. Patient Data

Tissue samples were obtained retrospectively from 82 GC patients who underwent gastric surgery with curative intent at Centro Hospitalar Universitário São João (CHUSJ; Porto, Portugal). Clinicopathological features were collected from medical and pathological records for further analysis (Supplementary Table S1). Formalin-fixed paraffin-embedded (FFPE) specimens from 41 intestinal-type, 15 diffuse-type, 11 mixed-type, and 15 indeterminate-type GC tumors, according to Laurén histological classification, were included. For each case, the H&E-stained section used for histological classification was sequential to the one used for immunophenotyping analysis, to allow the association between the T cell immune profiling and tissue histological features. All cases were staged according to the eighth edition of the American Joint Committee on Cancer (AJCC) staging system. None of the patients included in this study was treated with neoadjuvant chemotherapy nor chemo-radiation therapy. The study was approved by the CHUSJ Ethics Committee (protocol 78/13).

### 4.2. Immunofluorescence Staining, Image Acquisition, and Analysis

T cell immunophenotyping of GC samples was performed using a seven-target immunofluorescence detection protocol on single FFPE tissue sections, following a previously described combination of tyramide signal amplification (TSA) with direct and indirect immunostaining [36]. Briefly, after an initial deparaffinisation of 4-µm tissue sections with xylene and washing with ethanol, endogenous peroxidase was inactivated by blocking with 0.3% hydrogen peroxide in methanol (20 min; Merck Millipore, Burlington, MA, USA) and heat-induced antigen retrieval was performed with Tris-EDTA buffer (10 mM/1 mM, pH 9.0, 10 min). After cooling, slides were incubated with Superblock solution (30 min; Thermo Fisher Scientific, Waltham, MA, USA) to block non-specific binding. For TSA, slides were incubated with anti-PD-1 antibody (D4W2J, 1:2000 dilution, 60 min; Cell Signalling Technology, Danvers, MA, USA), followed by incubation with poly-horseradish peroxidase solution (30 min; Immunologic, Duiven, The Netherlands) and with Opal 520 reagent (1:100 dilution, 60 min; Perkin Elmer, Waltham, MA, USA). Before proceeding to the indirect immunofluorescence step, microwave-induced antibody stripping was achieved by incubating slides in citrate buffer (10 mM, pH 6.0) at 90 W for 15 min. For the two-step immunofluorescence detection, slides were incubated with anti-FoxP3 (236A/E7, 1:25 dilution; Thermo Fisher Scientific), anti-CD8 (4B11, 1:50 dilution; Dako, Glostrup, Denmark) and anti-CD103 (EPR4166 2, 1:100 dilution; Abcam, Cambridge, UK) primary antibodies overnight, and with corresponding conjugated secondary antibodies (CF and Alexa Fluor dyes; Sigma–Aldrich, Saint Louis, MO, USA, and Thermo Fisher Scientific, respectively) for 60 min. Direct conjugated antibodies anti-CD3-AF594 (EP447E; Abcam) and anti-keratin-AF546 (AE1/AE3 and C11; Thermo Fisher Scientific and Cell Signalling Technology) were incubated for 5 h, after which slides were incubated with DAPI (1 µM) for nuclear staining and mounted using Prolong™ gold antifade reagent (Cell Signalling Technology). Further details on the antibodies used can be found in Supplementary Table S2.

A complete scan of the slides was obtained using Vectra 3.0 Automated Quantitative Pathology Imaging System (4× magnification; Perkin Elmer) after which three regions of interest at the tumor core and another three at the invasive front were scanned with 20× magnification (1340 × 1000 µm images). Single-marker immunostainings were used to create the spectral libraries at the InForm Cell Analysis software (Perkin Elmer; Supplementary Figure S8A). DAPI/keratin staining was used to segment images into tumor nest and stroma (Supplementary Figure S8B). For cell segmentation, DAPI staining was used to segment nuclei, while CD3/CD8 staining was used to detect cell membrane (Supplementary Figure S8C). The resulting cells were analyzed based on the following phenotypes (Supplementary Figure S8D): (1) T helper cells, which were CD3^+^CD8^-^FoxP3^-^ cells; (2) CD8 T cells, CD3^+^CD8^+^; (3) and Treg cells that were CD3^+^FoxP3^+^ but CD8^-^. We have also analyzed the expression of CD103 (resident T cells) and PD-1 (activated cells) within these phenotypes (Supplementary Figure S8E). This data was not included in the main manuscript; however, it is available in Supplementary File S1. Cells expressing cytokeratin were excluded when building T cell profiles. For each of the previous steps (i.e., tissue segmentation, cell segmentation, and immunophenotyping), the InForm Cell Analysis software was manually trained before automated classification. Cell density (cells/mm^2^) was calculated by normalizing the cell counts by the tissue area of a given image.

### 4.3. Gastric Cancer 3D Cell Culture

Human GC cell lines MKN74 and MKN45 (intestinal- and diffuse-type GC, respectively; Japanese Collection of Research Bioresources Cell Bank) were seeded on 3D CoSeedis matrices (1 × 10^5^ cells/mL; abc biopply, Solothurn, Switzerland), as previously described [33], and cultured in RPMI 1640 medium (Thermo Fisher Scientific) supplemented with 10% fetal bovine serum (FBS; Biowest, Nuaillé, France) and 1% penicillin-streptomycin (PS; Thermo Fisher Scientific), at 37 °C in 5% CO_2_ humidified atmosphere, for 7 days to allow spheroid formation before co-culture with T cells.

### 4.4. Preparation of Treg and Conventional T Cell Enriched Samples

Human T cells were freshly isolated from anonymized healthy donor buffy coats, provided by Instituto Português do Sangue e da Transplantação (Porto, Portugal). The isolation of immune cells from healthy blood donors was approved by the CHUSJ Ethics Committee (protocol 90/19) after each donor informed consent collection. Blood samples were diluted (1:3) in 2% FBS/PBS, layered on top of Histopaque^®^-1077 (Sigma-Aldrich, St. Louis, MO, USA), on SepMate^TM^ tubes (Stemcell Technologies, Vancouver, BC, Canada), and centrifuged at 1200× *g* (10 min, with the brake on). Resulting peripheral blood mononuclear cells (PBMCs) were enriched for CD4^+^ T cells using MojoSort^TM^ Human CD4 T Cell Isolation Kit (BioLegend, San Diego, CA, USA). According to the manufacturer instructions, PBMCs were filtered through a 70-µm cell strainer and incubated with CD4 magnetic Nanobeads (20 min, on ice). CD4+ T cells were separated and washed in 2% FBS/PBS using a DynaMag™-15 magnet (Thermo Fisher Scientific). Thereafter, CD4^+^ T cells were incubated with fluorescent conjugated antibodies anti-CD3-PE (OKT3, 1:400 dilution; Thermo Fisher Scientific), anti-CD4-FITC (OKT4, 1:400 dilution; Thermo Fisher Scientific), anti- IL2Rα-PE/Cy7 (BC96, 1:25 dilution; Thermo Fisher Scientific) and anti-CD127 APC/Cy7 (eBioRDR5, 1:50 dilution; Thermo Fisher Scientific) for 30 min. Fluorescence-activated cell sorting (FACS) of Tregs (CD3^+^CD4^+^CD127^−^IL2Rα^+^) and conventional T cells (CD3^+^CD4^+^CD127^+^IL2Rα^−^) was performed on FACSAriaII flow cytometer (BD Biosciences). Further details on the antibodies used can be found in Supplementary Table S2.

### 4.5. Co-Culture of GC Spheroids and T Cells

For the co-culture of GC spheroids and T cells, MKN74 (intestinal-type) and MKN45 (diffuse-type) GC spheroids were transferred to round-bottom 96-well plates, and Tregs/conventional T cells were added to the culture media at 1:1, 1:5, and 1:15 (GC cell:T cell) ratios. Co-cultures were maintained in RPMI 1640 with 25 mM HEPES (Lonza, Basel, Switzerland) supplemented with 10% FBS, 1% PS, 1% Sodium pyruvate (Sigma-Aldrich) and recombinant human IL-2 (10 ng/mL; PeproTech, London, UK). Treg cultures were further supplemented with anti-CD3/anti-CD28 MACSiBead (0.5 beads/T cell; T Cell Activation Kit, Miltenyi Biotec, Bergisch Gladbach, Germany). As a control, MKN74/MKN45 single cultures (1:0; GC cell:T cell) were maintained in the same conditions as co-cultures. For all co-culture experiments (Figure 3, Figure 4 and Figure 5), a minimal number of 6 independent T cells donors, collected and analyzed in at least 3 independent days are represented.

### 4.6. Live Cell Imaging

To monitor T cell infiltration into tumor spheroids, MKN74 and MKN45 cell lines stably expressing mEmerald were used to form the GC spheroids, and sorted Tregs and conventional T cells were stained with CellTrace™ Far Red (CTFR) Cell Proliferation Kit (20 min, 37 °C; Thermo Fisher Scientific) before co-culture. First, co-cultures were imaged using an automated fluorescence widefield HCS microscope (IN Cell Analyzer 2000; GE Healthcare, Chicago, IL, USA), equipped with a Plan-Fluor Nikon 20×/0.45 objective lens and a large-chip CCD camera (CoolSNAP K4). Emerald-GC spheroids were acquired in the FITC channel (Excitation/Emission: 490/ 525 nm; Exposure: 50 ms) and T cells in the Cy5 channel (Excitation/Emission: 645/705 nm; Exposure: 30 ms). Images were acquired at every 2 h for 60 h, under temperature-controlled conditions.

To further confirm Treg (CTFR) infiltration into intestinal-type GC spheroids (MKN74-Emerald), spheroids were transferred to a fluorinated ethylene propylene (FEP) microtube and imaged with a custom-built Digital Scanner Laser Light Sheet microscope (LSFM) equipped with a Nikon Plan-Fluor 10x/0.3 water-immersion objective lens in illumination and detection plans. The fluorescence signals of the Emerald-MKN74 spheroids and CTFR-Tregs were recorded using a Hamamatsu Orca-Flash 4.0 V3 camera, after sample excitation with a 488 (1.0 mW) and 640 nm (1.0 mW) laser lines, respectively, with a 525/50 nm bandpass (BP) emission filter for emerald-spheroids and a 700/75 nm BP emission filter for CTFR-Tregs. The co-culture was imaged at three timepoints (16 h, 24 h, and 48 h) from four angles (90° rotation) every 16 min. Each view consists of multiple slices 1 µm apart covering the entire spheroid. Image reconstruction was performed using the arivis Vision4D v3.1.4 (arivis AG, Rostock, Germany).

### 4.7. Immunophenotype by Conventional and Imaging Flow Cytometry Analysis

After 48 h of co-culture, conditioned media and spheroids were collected to analyze the phenotype of Tregs and conventional T cells. Before co-culture with GC spheroids, Tregs and conventional T cells were stained using CellTrace™ Violet (CTV) Cell Proliferation Kit (20 min, 37 °C; Thermo Fisher Scientific), to allow further evaluation of T cell proliferation and to separate T cells (CTV+) from GC cells (CTV-) in the flow cytometry analysis. To prepare single-cell suspensions, spheroids were dissociated using 0.5% Trypsin-EDTA (5 min, 37 °C; Thermo Fisher Scientific). Both T cells and GC cells were stained with anti-CD3-PE, anti-CD4-FITC, and anti-IL2Rα-PE/Cy7, as previously mentioned, and with Fixable Viability Dye eFluor™780 (1:1000 dilution; Thermo Fisher Scientific) to assess cell viability. Intracellular staining of FoxP3 was performed using the Foxp3/Transcription Factor Staining Buffer Set (Thermo Fisher Scientific), following manufacturer’s instructions. Briefly, cells were fixed with Fixation/Permeabilization Buffer (30 min; Thermo Fisher Scientific), blocked with human IgG (1 mg/mL, 15 min; Sigma-Aldrich) and stained with anti-FoxP3-APC (1:15, 30 min; Thermo Fisher Scientific). Data acquisition was performed on an LSRFortessa cytometer (Becton, Dickinson & Company, Franklin Lakes, NJ, USA) and analyzed using FlowJo v10 software (Becton, Dickinson & Company) following the gating strategy explained on Supplementary Figure S3A.

Imaging flow cytometry was performed on single-cell suspensions derived from co-cultures of MKN74-GC spheroids with Tregs/conventional T cells and stained with anti-IL2Rα-PE/Cy7 antibody. Data was acquired on an imaging flow cytometer (ImageStreamX; Amnis Corporation, Seattle, WA, USA) equipped with INSPIRE software. Samples were excited with a 488 nm argon laser, and for each event, bright-field cell images were acquired (40× magnification) on channel 1, while IL2Rα signal was detected on channel 6 (Excitation/Emission: 496/774 nm). Laser power was not modified throughout the sample acquisition. Data analysis was performed using the IDEAS software (Amnis Corporation). Further details on the antibodies used can be found in Supplementary Table S2.

### 4.8. Recovery of IL2Rα^+^ GC Cells and Western Blot Analysis

To analyze the effect of IL2Rα expression specifically on intestinal-type GC cells, MKN74-GC cells were stained with CellTrace™ Yellow (CTY) Cell Proliferation Kit (Thermo Fisher Scientific) before grown as spheroids, while Tregs and conventional T cells were stained with CellTrace™ Far Red (CTFR) Cell Proliferation Kit (Thermo Fisher Scientific) before co-culture. As previously, after co-culture, spheroids were dissociated, and the resulting single-cell suspension was stained with anti-IL2Rα-PE/Cy7 antibody. MKN74-GC cells (CTY+) positive or negative for IL2Rα were sorted on the FACSAria II flow cytometer (Becton, Dickinson & Company) and collected to FBS. According to the number of sorted cells, samples were resuspended in proportional volumes of radioimmunoprecipitation assay buffer (RIPA buffer) supplemented with protease and phosphatase inhibitors (Thermo Fisher Scientific) and cell lysis was allowed for 30 min, on ice. To enhance protein recovery, lysates were sonicated twice (10 s, 20×, 50%) before centrifugation at 14,000 rpm (15 min, 4 °C) to recover cleared lysates. Equal amounts of protein lysate (25 µL) from each biological replicate were subjected to Western blotting. Primary antibodies targeting phospho-Stat3 (Tyr705, D3A7; Cell Signalling Technology), Stat3 (124 h6; Cell Signalling Technology), phospho-Akt (Ser473, D9E; Cell Signalling Technology), Akt (Cell Signalling Technology), phospho-p44/42 MAPK (phospho-ERK1/2, Thr202/Tyr204; Cell Signalling Technology), p44/42 MAPK (ERK1/2; Cell Signalling Technology), and GAPDH (1E6D9; ProteinTech, Rosemont, IL, USA) were used, as well as corresponding ECLTM anti-mouse and anti-rabbit IgG horseradish peroxidase-conjugated secondary antibodies (GE Healthcare). Detected signals were quantified using Quantity One^®^ Basic software (Bio-Rad, Hercules, CA, USA). Unprocessed scans of Western blots are provided in Supplementary Figure S9. Additional information on the antibodies used is detailed in Supplementary Table S2.

### 4.9. Ki-67 Staining on GC-Spheroids

To investigate GC-spheroid proliferation after co-culture, spheroids were fixed with 2% paraformaldehyde (overnight; Merck, Darmstadt, Germany), stained with Gill’s hematoxylin (10 min; Bio-Optica, Milan, Italy) and injected within a drop of 2.4% low melting point agarose (50 °C; Lonza). After gelling (10 min at room temperature followed by 20 min on ice), the agarose structures were included in paraffin blocks and then sectioned into 3-µm slides. Antigen retrieval was performed using citrate buffer (10 mM, pH 6.0, at 98 °C, 40 min; Abcam). Endogenous peroxidase activity was blocked using 0.3% hydrogen peroxidase solution (20 min; Sigma-Aldrich) followed by incubation with anti-Ki-67 antibody (SP6, 1:200 dilution, 90 min; Thermo Fisher Scientific). After washing, slides were incubated with REAL EnVision Detection System (Dako) substrate buffer (30 min) and with DAB Chromogen (10 min).

## 5. Conclusions

Overall, our study suggests that during the early steps of intestinal-type gastric carcinogenesis, Tregs accumulate within the tumor microenvironment and, likely through a contact-dependent mechanism, promote IL2Rα expression and stimulation of growth signaling pathways, such as MAPK pathway, in tumor cells. Our findings hold promising and relevant implications for future IO therapies and patient stratification in GC, which may potentially be expanded to other immune cold tumors.

## Data Availability

Data is contained within the article and/or supplementary material. Additional data presented in this study are available on request from the corresponding author.

## Supplementary Materials

The following are available online at https://www.mdpi.com/2072-6694/13/3/421/s1, Figure S1: Distribution of T cell populations in MSS and MSI GC tumours, Figure S2: Gating strategy of Tregs and conventional T cell sorting from peripheral blood of healthy donors, Figure S3: T cell infiltration of GC spheroids by light-sheet microscopy, Figure S4: Characterization of T cells before and after co-culture, Figure S5: Effect of IL2, anti-CD3/anti-CD28 beads, and co-culture conditioned medium in the expression of IL2Rα in GC cells., Figure S6: GC cell phenotype after co-culture with T cells, Figure S7: Distribution of T cell populations in intestinal-type GC patients and association with clinicopathological features, Figure S8: Step-wise multispectral imaging analysis of the immune T cell landscape in GC tissue sections, Figure S9: Western blot quantification for phosphorylated (p-ERK1/2) and total ERK1/2 protein levels in intestinal-type IL2Rα- and IL2Rα+ GC cells after 48 h co-culture with Tregs, Table S1: Description of patient information included in the analysed GC cohort, Table S2: Detailed description of antibodies, Videos S1–S3: Tregs (red) infiltration of intestinal-type GC spheroids (green) after 16 h (Supplementary Video S1), 24 h (Supplementary Video S2) and 48 h (Supplementary Video S3) of co-culture at 1:5 (GC:T) cell proportion, by light-sheet microscopy, File S1: Tumour presents lymphoid stroma (histological analysis).
