# Supplementary material for: Immunophenotype of Gastric Tumors Unveils a Pleiotropic Role of Regulatory T Cells in Tumor Development
Source: Cancers (Basel). 2021 Jan 23;13(3):421. doi: 10.3390/cancers13030421 (PMC7865950; doi:10.3390/cancers13030421)
Supplement: Supplementary file 1 [file cancers-13-00421-s001.zip › Supplementary videos/Supplementary videos/Supplementary_Video_Legends.docx]

**Supplementary Video 1-3.** Tregs (red) infiltration of intestinal-type GC spheroids (green) after 16h **(Supplementary Video 1)**, 24h **(Supplementary Video 2)** and 48h (**Supplementary Video 3)** of co-culture at 1:5 (GC:T) cell proportion, by light-sheet microscopy.
