# Supplementary material for: Immunophenotype of Gastric Tumors Unveils a Pleiotropic Role of Regulatory T Cells in Tumor Development
Source: Cancers (Basel). 2021 Jan 23;13(3):421. doi: 10.3390/cancers13030421 (PMC7865950; doi:10.3390/cancers13030421)
Supplement: Supplementary file 1 [file cancers-13-00421-s001.zip › Supplementary_Figures.pdf]

SUPPLEMENTARY FIGURES

Article title: Immunophenotype of gastric tumours unveils a pleiotropic role of regulatory T cells in tumour development

Journal name: Cancers

Authors: Sara Rocha, Afonso Basto, Marieke E Ijsselsteijn, Sara P Teles, Maria M Azevedo, Gilza Gonçalves, Irene Gullo, Gabriela M Almeida, Joaquín J Maqueda, Marta Oliveira, Fátima Carneiro, João T Barata, Luís Graça, Noel FCC de Miranda, Joana Carvalho and Carla Oliveira

Corresponding author:

Carla Oliveira

Group Coordinator, Expression Regulation in Cancer Group, i3S and Ipatimup - Institute of Molecular Pathology and Immunology, University of Porto

Professor of Oncobiology at the Faculty of Medicine of Porto University, Portugal

E-mail: carlaol@ipatimup.pt

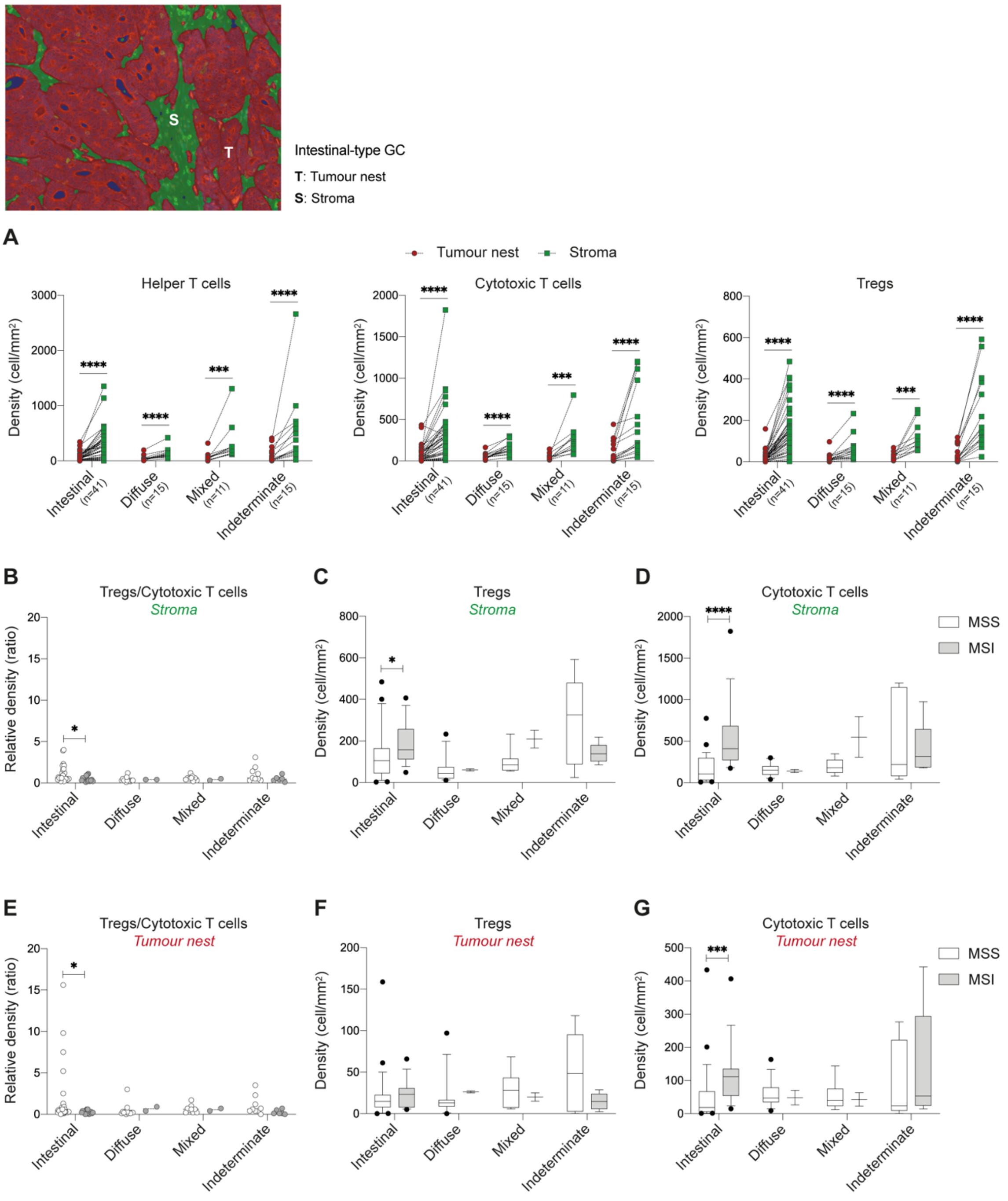

Supplementary Figure 1. Distribution of T cell populations in MSS and MSI GC tumours. (A) Density of helper, cytotoxic T cells and Tregs within tumour nest (red) and stroma (green) areas, according to tumour histology. Each dashed line matches tumour nest and stroma areas of the same patient sample. *p<0.05, ***p<0.001, ****p<0.0001; Wilcoxon matched-pairs signed-rank test. (B-G) Relative (B, E) and absolute numbers of Tregs (C, F) and cytotoxic T cells (D, G) within the stroma (B-D) and tumour nest (E-G) areas of intestinal-type GC cases, according to MSI status and tumour histology. Box and whiskers represent median ± 10–90 percentile. *p<0.05, **p<0.01, ***p<0.001, ****p<0.0001. Mann-Whitney U test (F-I).

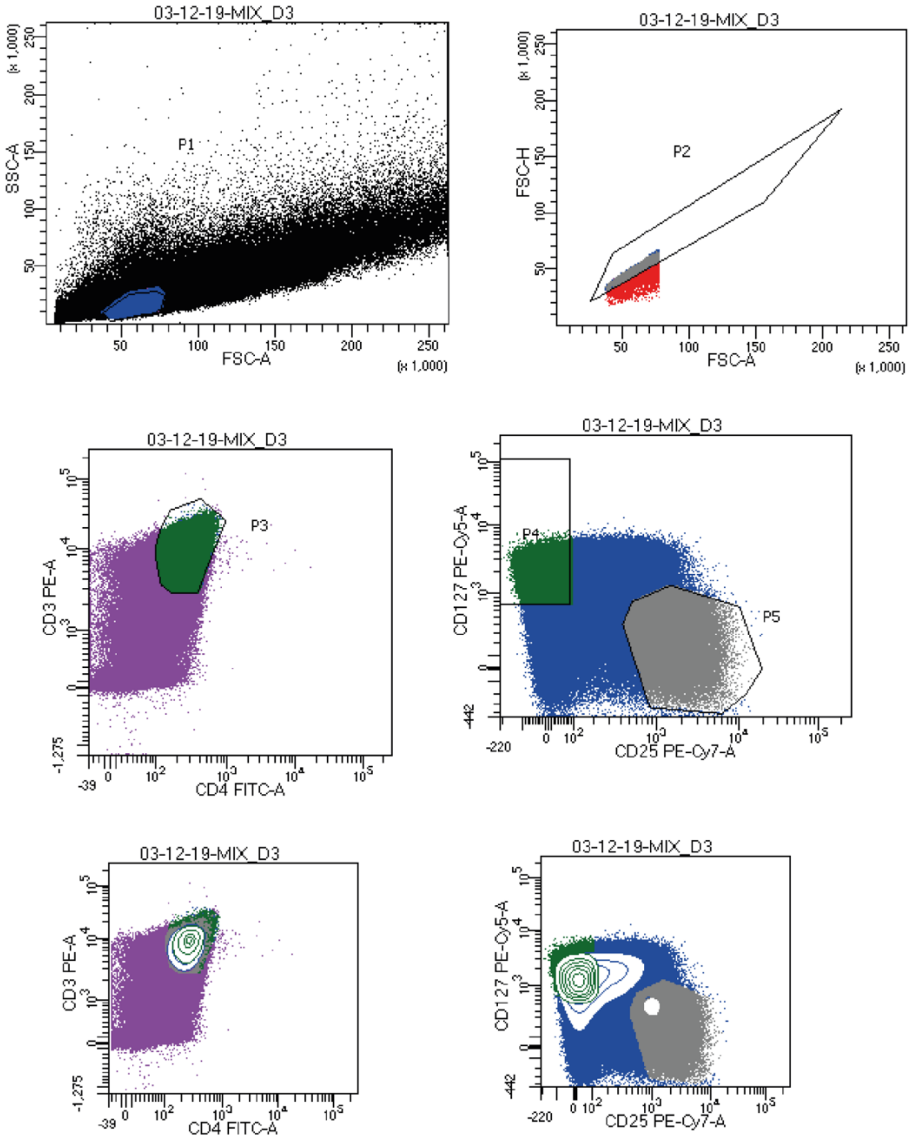

Tube: MIX_D3

Population	#Events	%Parent	%Total
All Events	1,957,727	####	100.0
P1	1,577,531	80.6	80.6
P2	1,566,396	99.3	80.0
P3	1,462,524	93.4	74.7
P4	732,511	50.1	37.4
P5	102,294	7.0	5.2

Population description:

P1 - T cells

P2 - Single cells

P3 - CD4 T cells (CD3+CD4+)

P4 - Conventional T cells (CD3+CD4+CD127+IL2Rα-)

P5 - Tregs (CD3+CD4+CD127-IL2Rα+)

Supplementary Figure 2. Gating strategy of Tregs and conventional T cell sorting from peripheral blood of healthy donors. Firstly, T cells (P1) are gated from the general events acquired, and single cells (P2) are selected from this population. Then, $CD3^{+}CD4^{+}$ T cells are gated (P3) followed by the identification of naïve T cells, $IL2R\alpha^{-}CD127^{+}$ (P4), and Tregs $IL2R\alpha^{+}CD127^{-}$ (P5).

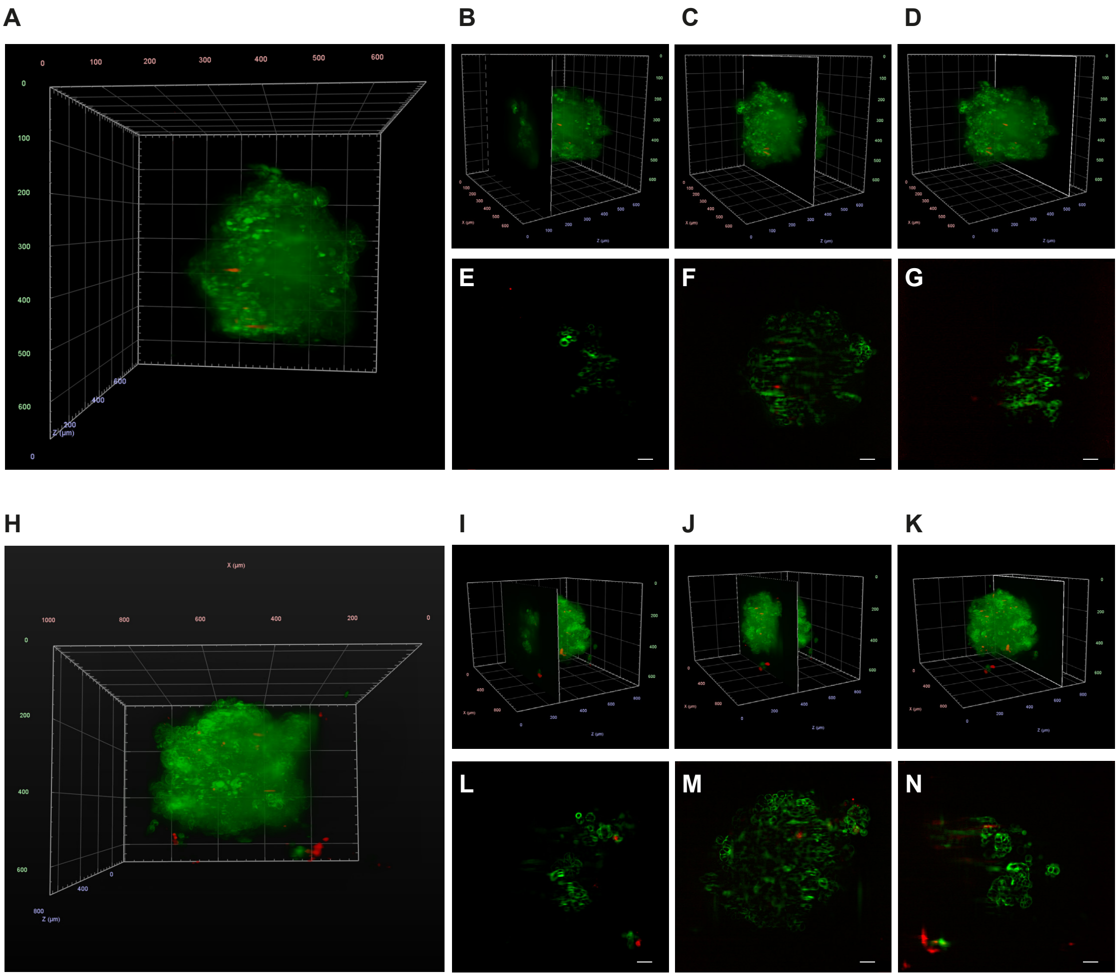

Supplementary Figure 3. T cell infiltration of GC spheroids by light-sheet microscopy. Co-cultures of intestinal-type GC spheroids (green) and Tregs (red) were imaged at 16h (A-G) and 48h (H-N). (A, H) 3D tomography of a four-angle fusion of the co-culture. Location of (B, I) section 1 (135 and 270 nm, respectively), (C, J) section 2 (348 and 392 nm, respectively) and (D, K) section 3 (552 and 678 nm, respectively) in relation to the entire co-culture. Images of cross-sections 1 (E, L), 2 (F, M) and 3 (G, N) show Tregs within GC spheroids. Scale bar: 50 μm.

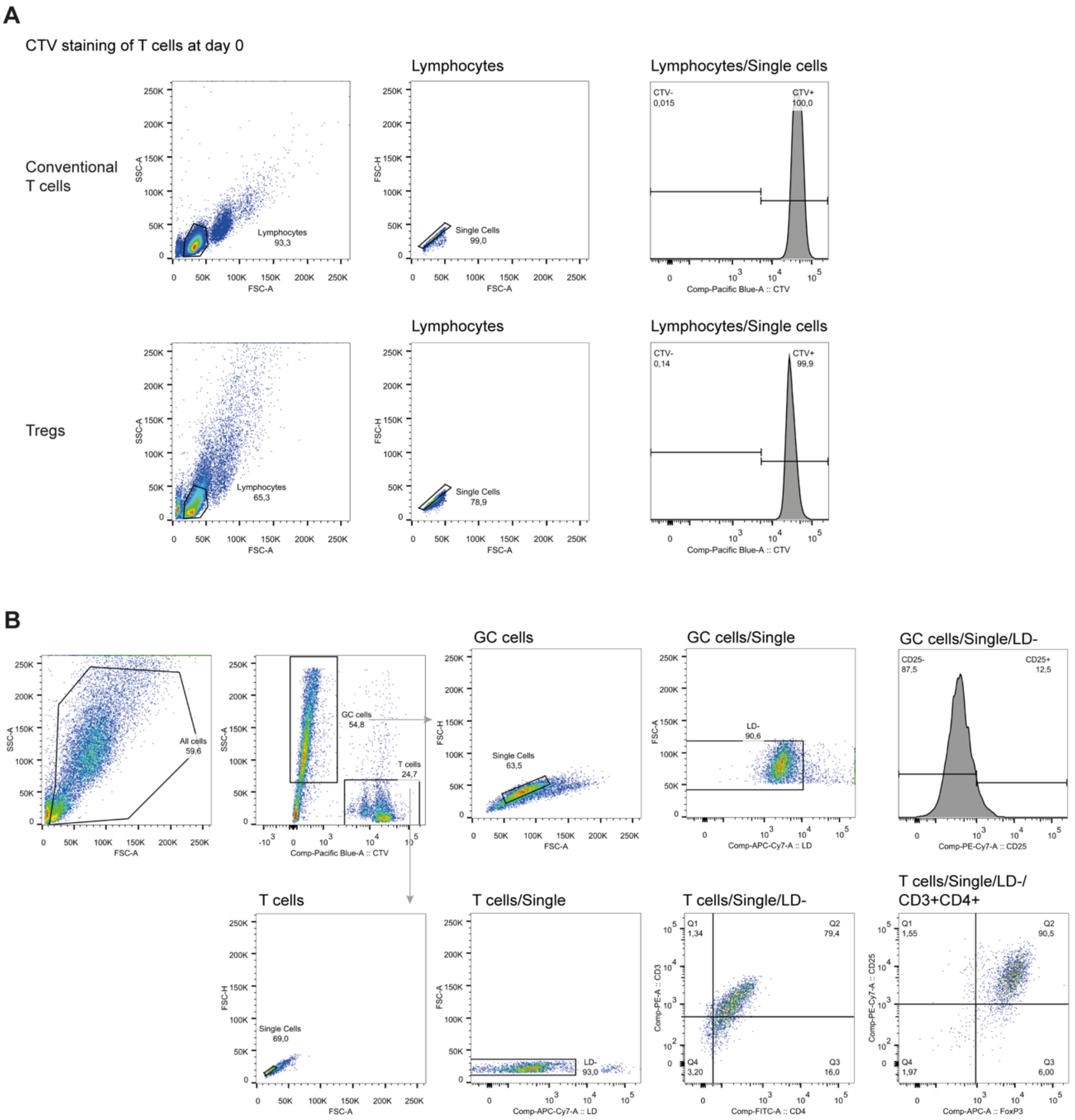

Supplementary Figure 4. Characterization of T cells before and after co-culture. (A) Detection of CTV on conventional T cells and Tregs before co-culture showing that all T cells were successfully stained. (B) Step-wise analysis of the flow cytometry data acquired after co-cultures. From the "All cells" gate, GC cells and T cells were separated according to their size (SSC-A) and intensity of CTV dye (which is only present in T cells) and analysed separately. Within each cell populations, gates on single and live (LD negative) cells were used to analyse the expression of CD3, CD4, IL2Rα and FoxP3 markers in T cells and GC cells. In GC cells, IL2Rα was the only marker with altered expression after co-culture.

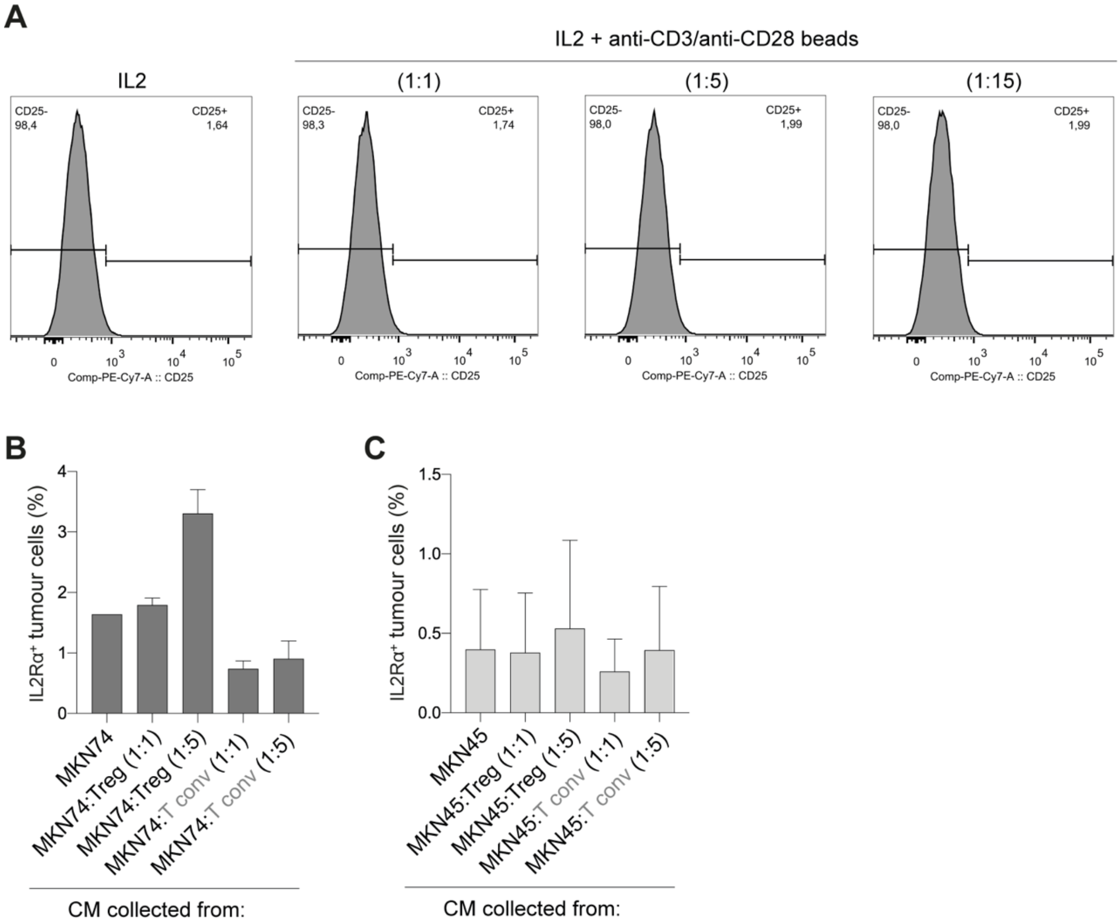

Supplementary Figure 5. Effect of IL2, anti-CD3/anti-CD28 beads, and co-culture conditioned medium in the expression of IL2Rα in GC cells. (A) Histograms showing that IL2Rα expression in intestinal-type GC cells is not altered by culture in IL2 only or IL2 with increasing concentrations of anti-CD3/anti-CD28 beads, the same concentrations used for the co-cultures with Treg cells. (C-D) Bar plots showing percentage of IL2Rα positive intestinal-type GC (B) or diffuse-type GC (C) cells after 48h co-culture with conditioned media collected from independent co-cultures with Tregs (dark grey) or conventinal T cells (light grey). Black bars represent spheroids that were not exposed to conditioned media from co-cultures. Data shown as mean±SD for co-cultures treated with T cells isolated from at least three healthy donors.

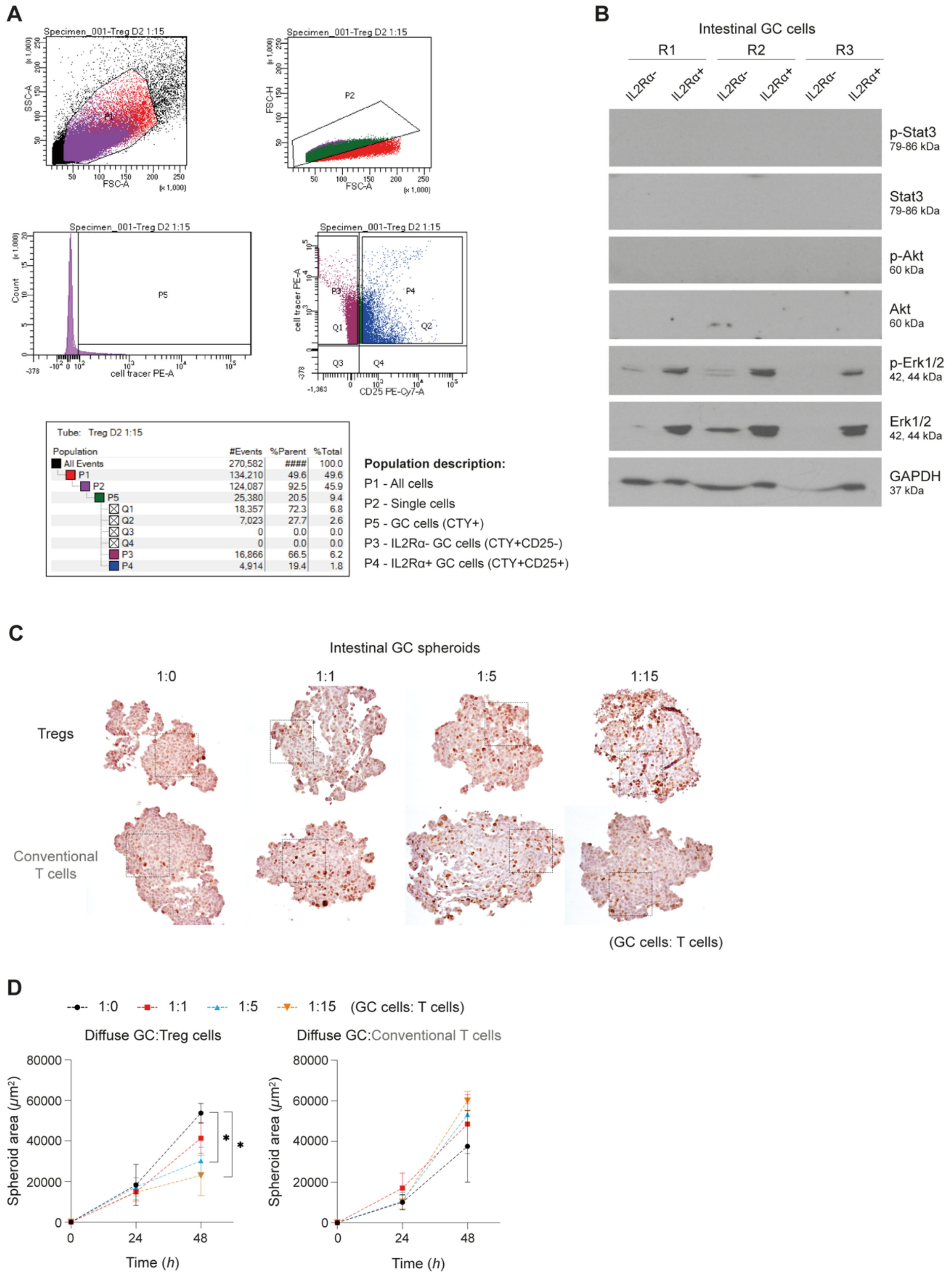

Supplementary Figure 6. GC cell phenotype after co-culture with T cells. (A) Gating strategy to analyse GC cells expressing IL2Rα at cell membrane. Intestinal-type GC cells were gated on general events acquired (P1), and single cells (P2) are selected from this population. Then, Cell Tracer Yellow^{+} cells (only GC cells) were gated (P5) followed by the identification of IL2Rα^{-} (P3) and IL2Rα^{+} (P4) GC cells. (B) Western blot analysis for phosphorylated and total protein levels of

Stat3, Akt and Erk1/2 in intestinal-type IL2Rα$^-$ and IL2Rα$^+$ GC cells after 48h co-culture with Tregs. Images show unprocessed scans for three biological replicates. (C) Whole spheroid image sections of Ki-67 nuclear expression in intestinal GC spheroids after 48h of co-culture with Treg or conventional T cells, at different 1:0 (control), 1:1, 1:5 or 1:15 proportions. Selected region are shown by the dash boxes. (D) Plots of growth area for intestinal GC spheroids co-cultured with Treg (left graph) or conventional T cells (right graph) for 48h. Control spheroids (dark) represent intestinal GC spheroids without T cells in co-culture. Co-cultures at 1:1, 1:5 and 1:15 proportions are represented in red, blue and orange respectively. Data shown as mean±SD of three independent co-cultures. *$p<0.05$. Two-way ANOVA with Dunnett's multiple comparisons test.

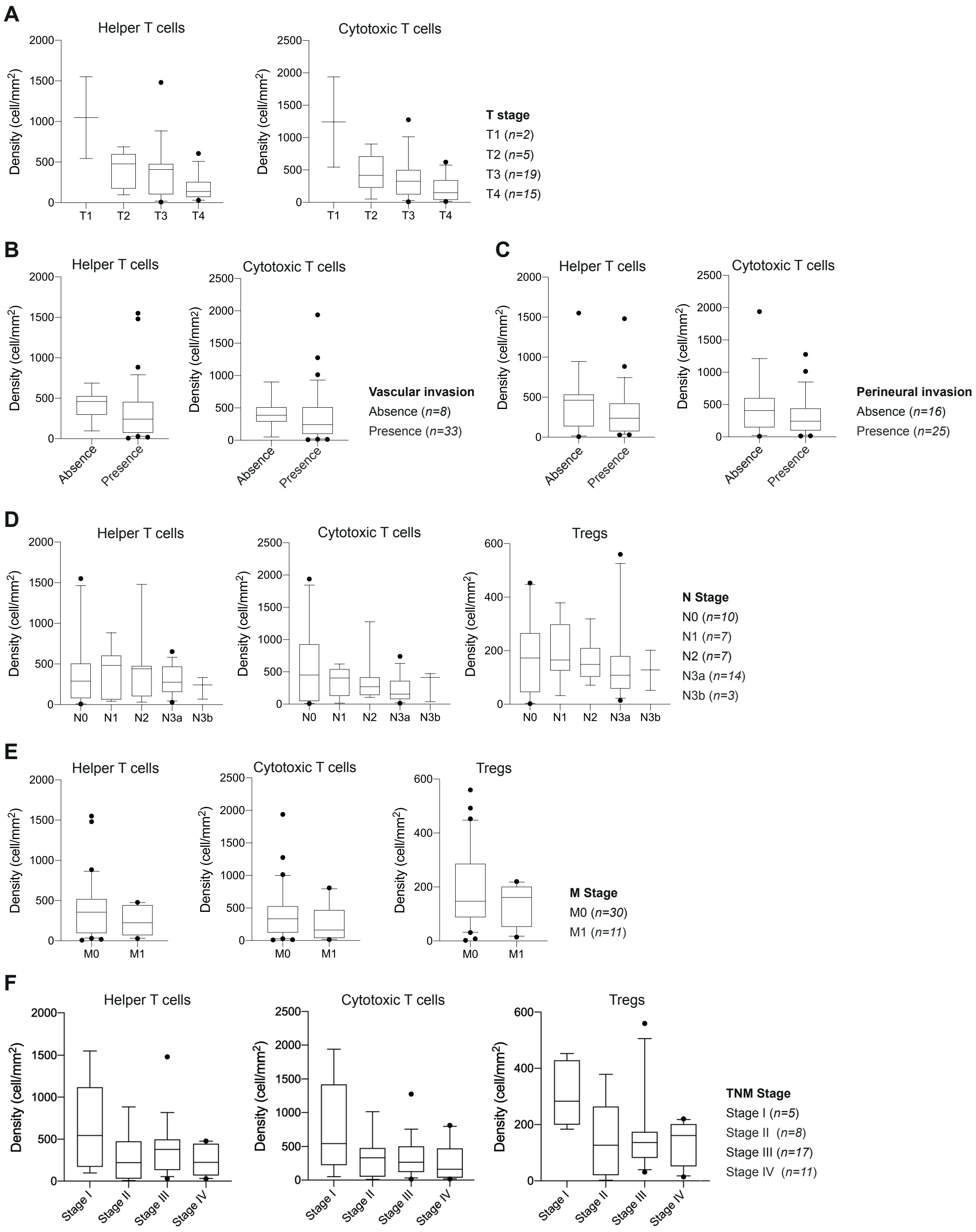

Supplementary Figure 7. Distribution of T cell populations in intestinal-type GC patients and association with clinicopathological features. (A-C) Density of helper and cytotoxic T cells according to (A) T stage: T1, n=2; T2, n=5; T3, n=19; T4, n=15; (B) absence (n=8) or presence (n=33) of vascular invasion; and, (C) absence (n=16) or presence (n=25) of perineural invasion. (D-F) Density of helper, cytotoxic T cells and Tregs according to (D) N stage: N0, n=10; N1, n=7; N2, n=7; N3a, n=14; N3b, n=3; (E) M stage: M0, n=30; M1, n=11; and, (F) TNM stage: Stage I, n=5; Stage II, n=8; Stage III, n=17; and, Stage IV, n=11. Box and whiskers represent median ± 10–90 percentile.

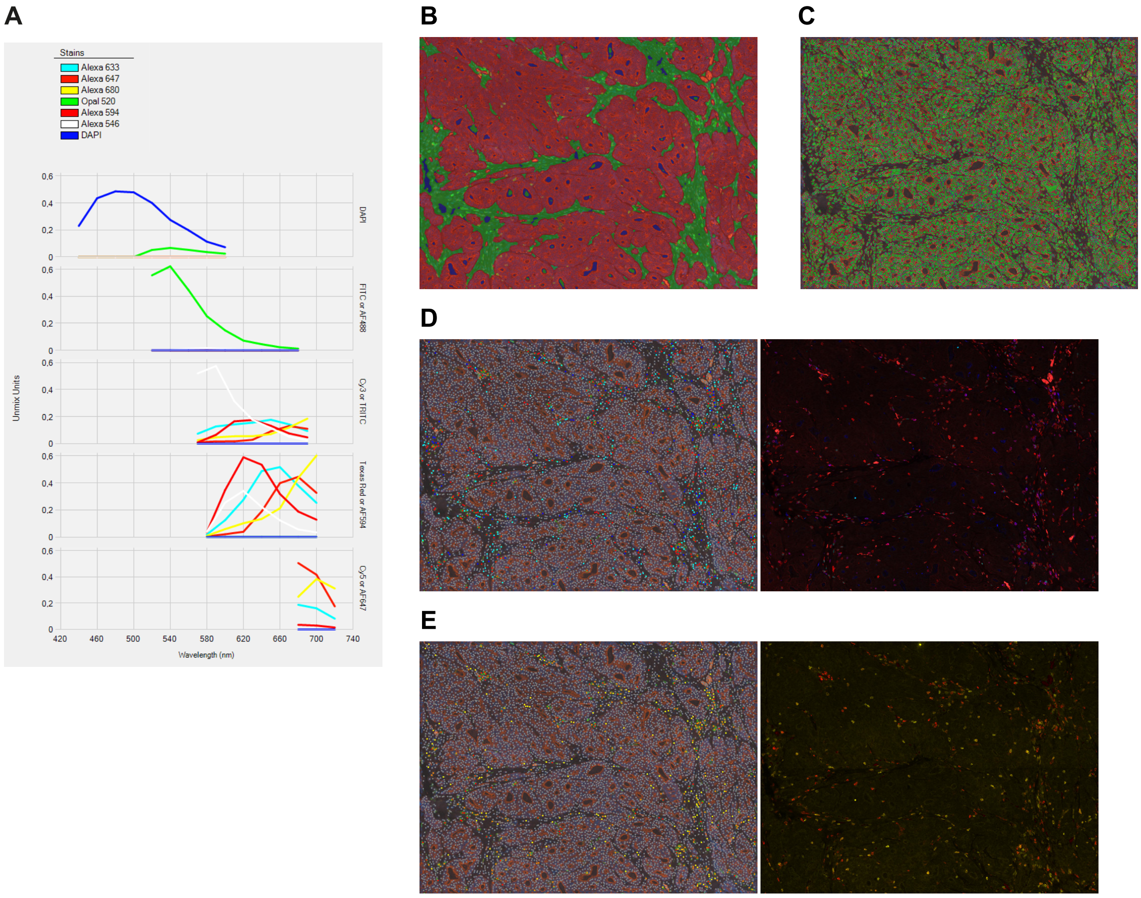

Supplementary Figure 8. Step-wise multispectral imaging analysis of the immune T cell landscape in GC tissue sections. (A) Spectral library used for dye separation and image preparation at InForm cell analysis software. (B-E) Images illustrating the consecutive steps for image analysis in InForm software. (B) Tissue segmentation into tumour nest (red), and stroma (green). Areas without nuclei detection are represented in blue. (C) Cell segmentation based on nuclei and membrane staining. (D-E) Phenotyping and automatic cell count. In a first analysis cell phenotype was based on CD3, CD8 and FoxP3 expression (D) and in a second analysis, based on CD103 and PD1 expression (E).

A

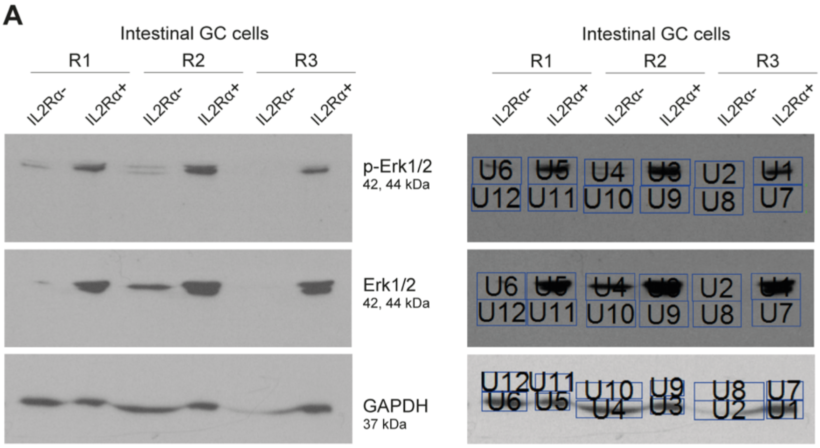

B

	GAPDH		ERK		p-ERK	
	**Density**	**Area**	**Density**	**Area**	**Density**	**Area**
**U1**	19870,07	14,91	19252,26	30,28	17037,66	30,28
**U2**	15462,96	29,81	14456,41	30,28	15161,28	30,28
**U3**	20219,48	12,04	20297,92	30,28	18374,57	30,28
**U4**	19154,49	25,38	17093,08	30,28	15773,04	30,28
**U5**	19232,96	13,78	18330,32	30,28	17521,25	30,28
**U6**	19112,25	20,90	14337,15	30,28	15589,32	30,28
**U7**	13884,50	14,91	14454,10	30,28	15254,70	30,28
**U8**	13711,19	29,81	14321,17	30,28	15082,64	30,28
**U9**	13672,77	12,04	14412,15	30,28	15127,92	30,28
**U10**	13638,36	25,38	14204,42	30,28	15038,42	30,28
**U11**	13558,74	13,78	14050,85	30,28	15174,91	30,28
**U12**	13565,44	20,90	14077,44	30,28	15070,78	30,28

C

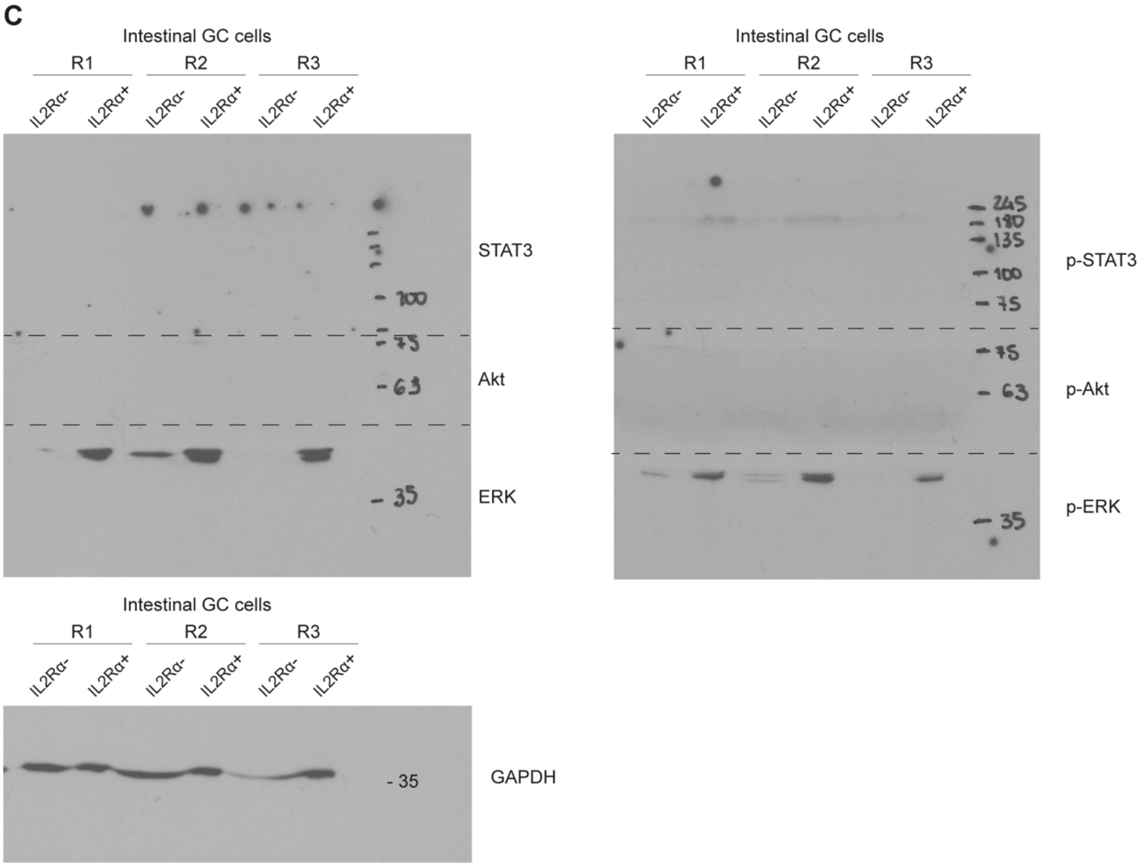

Supplementary Figure 9. Western blot quantification for phosphorylated (p-ERK1/2) and total ERK1/2 protein levels in intestinal-type IL2Rα$^-$ and IL2Rα$^+$ GC cells after 48h co-culture with Tregs. (A) Images showing unprocessed scans for three biological replicates (left panel) and band quantification strategy (right panel). (B) Densitometry readings and areas of each band used for quantification. (C) Whole blot imagens showing all the bands and molecular weight markers. Dashed lines indicate were membranes have been cut to stain simultaneously for STAT3, Akt and ERK total or phosphorylated forms.
