# Supplementary material for: Immunophenotype of Gastric Tumors Unveils a Pleiotropic Role of Regulatory T Cells in Tumor Development
Source: Cancers (Basel). 2021 Jan 23;13(3):421. doi: 10.3390/cancers13030421 (PMC7865950; doi:10.3390/cancers13030421)
Supplement: Supplementary file 1 [file cancers-13-00421-s001.zip › Supplementary_Tables.pdf]

**SUPPLEMENTARY TABLES**

**Article title**: Immunophenotype of gastric tumours unveils a pleiotropic role of regulatory T cells in tumour development

**Journal name:** Cancers

**Authors:** Sara Rocha, Afonso Basto, Marieke E Ijsselsteijn, Sara P Teles, Maria M Azevedo, Gilza Gonçalves, Irene Gullo, Gabriela M Almeida, Joaquín J Maqueda, Marta Oliveira, Fátima Carneiro, João T Barata, Luís Graça, Noel FCC de Miranda, Joana Carvalho and Carla Oliveira

**Corresponding author:**

Carla Oliveira

*Group Coordinator, Expression Regulation in Cancer Group, i3S and Ipatimup - Institute of Molecular Pathology and Immunology, University of Porto*

*Professor of Oncobiology at the Faculty of Medicine of Porto University, Portugal*

*E-mail: carlaol@ipatimup.pt*

**Supplementary Table 1.** Description of patient information included in the analysed GC cohort

| | |
|---|---|
| **Patient gender** | |
| Female | 37 *(45%)* |
| Male | 45 *(55%)* |
| **Age of onset** | |
| Mean | 70 |
| Standard deviation | 12 |
| **Histological type (Laurén Classification)** | |
| Intestinal | 41 *(50%)* |
| Diffuse | 15 *(18%)* |
| Mixed | 11 *(13%)* |
| Indeterminate | 15 *(18%)* |
| **TNM Staging** | |
| I | 8 *(10%)* |
| II | 13 *(16%)* |
| III | 42 *(51%)* |
| IV | 19 *(23%)* |
| **pT stage** | |
| T1b | 3 *(4%)* |
| T2 | 8 *(10%)* |
| T3 | 36 *(44%)* |
| T4a | 31 *(38%)* |
| T4b | 4 *(5%)* |
| **N Stage** | |
| N0 | 16 *(20%)* |
| N1 | 11 *(13%)* |
| N2 | 16 *(20%)* |
| N3a | 24 *(29%)* |
| N3b | 15 *(18%)* |
| **M stage** | |
| M0 | 63 *(77%)* |
| M1 | 19 *(23%)* |
| **Lymphatic Invasion** | |
| Absent | 8 *(10%)* |
| Present | 74 *(90%)* |
| **Vascular Invasion** | |
| Absent | 18 *(22%)* |
| Present | 64 *(78%)* |
| **Perineural Invasion** | |
| Absent | 26 *(32%)* |
| Present | 56 *(68%)* |
| **Microsatellite Instability** | |
| MSS | 57 *(70%)* |
| MSI | 25 *(30%)* |

**Supplementary Table 2.** Detailed description of antibodies

| Application | Target | Clone | Dilution | Species | Isotype | Fluorochrome | Supplier | Detection method |
|---|---|---|---|---|---|---|---|---|
| *Immunofluorescence* | PD1 | D4W2J | 1:2000 | Rabbit | IgG | Opal 520 | Cell Signaling Technology | Opal detection |
| | FoxP3 | 236A/E7 | 1:25 | Mouse | IgG1 | CF633 | Thermo Fisher Scientific | Indirect detection |
| | CD8 | 4B11 | 1:50 | Mouse | IgG2b | AF647 | Leica biosystems | Indirect detection |
| | CD103 | EPR4166 2 | 1:100 | Rabbit | IgG | AF680 | Abcam | Indirect detection |
| | CD3 | EP447E | 1:50 | Rabbit | IgG | AF594 | Abcam | Conjugated antibodies |
| | Cytokeratin | AE1/AE3 + C11 | 1:50 | Mouse | IgG1 | AF546 | Thermo Fisher Scientific and Cell Signaling Technology | Conjugated antibodies |
| *Flow Cytometry* | CD3 | OKT3 | 1:400 | Mouse | IgG2a | PE | Thermo Fisher Scientific | Conjugated antibodies |
| | CD4 | OKT4 | 1:400 | Mouse | IgG2b | FITC | Thermo Fisher Scientific | Conjugated antibodies |
| | CD127 | eBioRDR5 | 1:50 | Mouse | IgG1 | PE/Cy5 | Thermo Fisher Scientific | Conjugated antibodies |
| | CD25 | BC96 | 1:25 | Mouse | IgG1 | PE/Cy7 | Thermo Fisher Scientific | Conjugated antibodies |
| | FoxP3 | PCH101 | 1:15 | Rat | IgG2a | APC | Thermo Fisher Scientific | Conjugated antibodies |
| *Western Blotting* | phospho-Stat3 | Tyr705, D3A7 | 1:1000 | Rabbit | IgG | NA | Cell Signaling Technology | Indirect detection |
| | Stat3 | 124H6 | 1:1000 | Mouse | IgG2a | NA | Cell Signaling Technology | Indirect detection |
| | phospho-Akt | Ser473, D9E | 1:1000 | Rabbit | IgG | NA | Cell Signaling Technology | Indirect detection |
| | Akt | NA | 1:1000 | Rabbit | NA | NA | Cell Signaling Technology | Indirect detection |
| | phospho-p44/42 MAPK | Thr202/Tyr204 | 1:1000 | Rabbit | NA | NA | Cell Signaling Technology | Indirect detection |
| | p44/42 MAPK | NA | 1:1000 | Rabbit | NA | NA | Cell Signaling Technology | Indirect detection |
| | GAPDH | 1E6D9 | 1:5000 | Mouse | IgG2b | NA | ProteinTech | Indirect detection |

NA: Not applicable
